# Development and validation of QRISK3 risk prediction algorithms to estimate future risk of cardiovascular disease: prospective cohort study

**DOI:** 10.1136/bmj.j2099

**Published:** 2017-05-24

**Authors:** Julia Hippisley-Cox, Carol Coupland, Peter Brindle

**Affiliations:** 1 Division of Primary Care, University Park, Nottingham NG2 7RD, UK; 2 Bristol Primary Clinical Commissioning Group and The National Institute for Health Research Collaboration for Leadership in Applied Health Research and Care West (NIHR CLAHRC West) at University Hospitals Bristol NHS Foundation Trust, UK, UK

## Abstract

**Objectives** To develop and validate updated QRISK3 prediction algorithms to estimate the 10 year risk of cardiovascular disease in women and men accounting for potential new risk factors.

**Design** Prospective open cohort study.

**Setting** General practices in England providing data for the QResearch database.

**Participants** 1309 QResearch general practices in England: 981 practices were used to develop the scores and a separate set of 328 practices were used to validate the scores. 7.89 million patients aged 25-84 years were in the derivation cohort and 2.67 million patients in the validation cohort. Patients were free of cardiovascular disease and not prescribed statins at baseline.

**Methods** Cox proportional hazards models in the derivation cohort to derive separate risk equations in men and women for evaluation at 10 years. Risk factors considered included those already in QRISK2 (age, ethnicity, deprivation, systolic blood pressure, body mass index, total cholesterol: high density lipoprotein cholesterol ratio, smoking, family history of coronary heart disease in a first degree relative aged less than 60 years, type 1 diabetes, type 2 diabetes, treated hypertension, rheumatoid arthritis, atrial fibrillation, chronic kidney disease (stage 4 or 5)) and new risk factors (chronic kidney disease (stage 3, 4, or 5), a measure of systolic blood pressure variability (standard deviation of repeated measures), migraine, corticosteroids, systemic lupus erythematosus (SLE), atypical antipsychotics, severe mental illness, and HIV/AIDs). We also considered erectile dysfunction diagnosis or treatment in men. Measures of calibration and discrimination were determined in the validation cohort for men and women separately and for individual subgroups by age group, ethnicity, and baseline disease status.

**Main outcome measures** Incident cardiovascular disease recorded on any of the following three linked data sources: general practice, mortality, or hospital admission records.

**Results** 363 565 incident cases of cardiovascular disease were identified in the derivation cohort during follow-up arising from 50.8 million person years of observation. All new risk factors considered met the model inclusion criteria except for HIV/AIDS, which was not statistically significant. The models had good calibration and high levels of explained variation and discrimination. In women, the algorithm explained 59.6% of the variation in time to diagnosis of cardiovascular disease (R^2^, with higher values indicating more variation), and the D statistic was 2.48 and Harrell’s C statistic was 0.88 (both measures of discrimination, with higher values indicating better discrimination). The corresponding values for men were 54.8%, 2.26, and 0.86. Overall performance of the updated QRISK3 algorithms was similar to the QRISK2 algorithms.

**Conclusion** Updated QRISK3 risk prediction models were developed and validated. The inclusion of additional clinical variables in QRISK3 (chronic kidney disease, a measure of systolic blood pressure variability (standard deviation of repeated measures), migraine, corticosteroids, SLE, atypical antipsychotics, severe mental illness, and erectile dysfunction) can help enable doctors to identify those at most risk of heart disease and stroke.

## Introduction

The first QRISK model to estimate 10 year risk of cardiovascular disease was published in 2007.^1^ It was followed by an updated model (QRISK2) in 2008, which included ethnic origin and additional risk factors (type 2 diabetes, rheumatoid arthritis, atrial fibrillation, and chronic renal disease). Since then, QRISK2 has been updated annually and recalibrated to the latest version of the QResearch database^2^; the age range across which it applies has also been extended from 35-74 years to 25-84 years, type 1 diabetes has been included as a separate variable, smoking is assessed at five levels instead of two, and the Townsend score has been updated using the most recent values from the 2011 census. This helps to ensure that the algorithms reflect the changes in population characteristics (such as changes in prevalence of smoking, body mass index, or declining incidence of cardiovascular disease) and improvements in data quality (such as improved recording of risk factors and data linkage to Hospital Episode Statistics,^3^ which has increased ascertainment of cardiovascular events^4^). The QRISK algorithms have been validated by ourselves and others in independent groups of patients using UK primary care databases such as QResearch,^4^ Clinical Practice Research Datalink (CPRD),^4^ The Health Improvement Network (THIN),^5^ ^6^ ^7^ ^8^ ^9^ and clinical cohorts^10^ ^11^ ^12^ as well as in international populations.^13^ ^14^ Their use has been evaluated in observational studies,^15^ cost effectiveness evaluations,^16^ and clinical trials.^17^ ^18^

QRISK2 is now used across England’s health service (NHS England) and recommended in the NHS Quality and Outcomes Framework,^19^ guidance from the National Institute of Health and Care Excellence,^20^ and NHS Health Check.^21^ QRISK2 is also used in occupational health settings and internationally, with over two million hits on the QRISK website (www.qrisk.org). A new NICE guideline on lipid modification and cardiovascular risk assessment was published in 2014.^20^ This guideline highlighted a number of conditions associated with increased cardiovascular risk that may not be fully captured by QRISK2, including HIV/AIDS, stage 3 kidney disease, systemic lupus erythematosus (SLE), severe mental illness, and use of atypical antipsychotics or corticosteroids.^20^ These conditions are not specifically identified within QRISK2, which may result in underestimation of risk in the relevant patient groups. In addition, recently published research has highlighted increased cardiovascular risk and potential prognostic importance for erectile dysfunction,^22^ ^23^ ^24^ migraine,^25^ and blood pressure variability.^26^ We therefore derived and validated a new version of the algorithms, QRISK3, to determine whether these factors should be incorporated into the algorithms to improve estimation of cardiovascular risk for these patients.

## Methods

### Study design and data source

Using the QResearch database (version 41) we undertook a cohort study in a large population of primary care patients. We included all practices in England that had been using the EMIS computer system for at least one year and randomly allocated three quarters of practices to the derivation dataset and the remainder to a validation dataset. We identified an open cohort of patients aged 25-84 years registered with the practices between 1 January 1998 and 31 December 2015. Patients were excluded if they had no postcode related Townsend score (since these usually result from patients moving to newly built houses with new postcodes not being yet linked to deprivation data or from patients being homeless or not having a permanent residence), had pre-existing cardiovascular disease (on general practice records or linked hospital records), or were using prescribed statins at cohort entry. We determined an entry date to the cohort for each patient, which was the latest of the following: 25th birthday, date of registration with the practice plus one year, date on which the practice computer system was installed plus one year, or the study start date (1 January 1998). Patients were censored at the earliest date of the diagnosis of cardiovascular disease, death, deregistration with the practice, last upload of computerised data, or study end date (31 December 2015).

### Outcomes

Our outcome was cardiovascular disease, which was defined as a composite outcome of coronary heart disease, ischaemic stroke, or transient ischaemic attack. The QResearch database is linked at individual patient level to hospital admissions data (Hospital Episode Statistics), and mortality records obtained from the Office for National Statistics. The records are linked using a pseudonymised NHS number specific to the QResearch database. The recording of NHS numbers is valid and complete for 99.8% of patients with data on QResearch, 99.9% for ONS mortality records, and 98% for hospital admissions records.^3^ ^27^ We classified patients as having cardiovascular disease if there was a record of the relevant clinical code in either their general practice record, their linked hospital record, or their linked mortality record. We used Read codes to identify cardiovascular disease cases from the general practice record. The Read codes are listed in table 1 of the web appendix. We used ICD-10 (international classification of diseases, 10th revision) clinical codes to identify cases from hospital and mortality records except for the three years between 1 January 1998 and 31 December 2000, when ICD-9 was in use for mortality records. The ICD-10 codes used were G45 (transient ischaemic attack and related syndromes), I20 (angina pectoris), I21 (acute myocardial infarction), I22 (subsequent myocardial infarction), I23 (complications after myocardial infarction), I24 (other acute ischaemic heart disease), I25 (chronic ischaemic heart disease), I63 (cerebral infarction), and I64 (stroke not specified as haemorrhage or infarction). The corresponding ICD-9 codes used were 410, 411, 412, 413, 414, 434, and 436. General practice and linked mortality and Hospital Episode Statistics data were available until 31 December 2015. We used the earliest recorded date of cardiovascular disease on any of the three data sources as the outcome date.

**Table 1 tbl1:** Baseline characteristics of patients aged 25-84 years without cardiovascular disease and not using statins at study entry. Values are numbers (percentages) of patients unless stated otherwise

Characteristics	Derivation cohort			Validation cohort	
	Women n=4 019 956	Men n=3 869 847		Women n=1 360 457	Men n=1 310 841
Mean (SD) age (years)	43.3 (15.3)	42.6 (14.0)		43.3 (15.3)	42.6 (13.8)
Mean (SD) Townsend score	0.4 (3.2)	0.5 (3.3)		0.4 (3.3)	0.5 (3.3)
Body mass index recorded	2 926 402 (72.8)	2 476 175 (64.0)		996 752 (73.3)	852 521 (65.0)
Total cholesterol: HDL cholesterol ratio recorded	1 598 558 (39.8)	1 467 747 (37.9)		543 262 (39.9)	501 605 (38.3)
Systolic blood pressure recorded	3 327 445 (82.8)	2 644 682 (68.3)		1 146 039 (84.2)	922 967 (70.4)
≥2 systolic blood pressure readings recorded before baseline	3 123 821 (77.7)	2 338 902 (64.0)		1 072 667 (78.8)	813 373 (62.0)
Complete data recorded for body mass index, total cholesterol: HDL cholesterol, systolic blood pressure, and smoking status	1 145 256 (28.5)	952 618 (24.6)		389 774 (28.7)	330 073 (25.2)
Mean (SD) body mass index	25.4 (5.1)	25.9 (4.2)		25.4 (5.1)	25.9 (4.2)
Mean (SD) total cholesterol: HDL cholesterol ratio	3.7 (1.2)	4.4 (1.4)		3.6 (1.2)	4.4 (1.3)
Mean (SD) systolic blood pressure (mm Hg)	123.2 (18.2)	129.2 (16.3)		123.1 (18.1)	128.8 (16.2)
Mean (SD) systolic blood pressure variability*	9.3 (6.2)	9.9 (6.8)		9.3 (6.1)	9.9 (6.8)
Ethnic origin:					
Recorded	2 607 872 (64.9)	2 310 983 (59.7)		849 697 (62.5)	751 370 (57.3)
White or not recorded	3 564 651 (88.7)	3 435 408 (88.8)		1 218 391 (89.6)	1 171 281 (89.4)
Indian	77 683 (1.9)	81 805 (2.1)		23 146 (1.7)	26 479 (2.0)
Pakistani	39 541 (1.0)	46 948 (1.2)		10 919 (0.8)	14 787 (1.1)
Bangladeshi	31 930 (0.8)	42 111 (1.1)		8738 (0.6)	11 914 (0.9)
Other Asian	53 559 (1.3)	45 753 (1.2)		17 078 (1.3)	15 966 (1.2)
Black Caribbean	37 781 (0.9)	30 610 (0.8)		13 142 (1.0)	10 642 (0.8)
Black African	77 813 (1.9)	71 245 (1.8)		27 678 (2.0)	25 251 (1.9)
Chinese	33 767 (0.8)	23 730 (0.6)		8992 (0.7)	6098 (0.5)
Other	103 231 (2.6)	92 237 (2.4)		32 373 (2.4)	28 423 (2.2)
Smoking status:					
Smoking status recorded	3 418 296 (85.0)	3 005 756 (77.7)		1 168 932 (85.9)	1 035 425 (79.0)
Non-smoker	2 051 803 (51.0)	1 463 941 (37.8)		706 671 (51.9)	512 252 (39.1)
Former smoker	589 521 (14.7)	594 265 (15.4)		194 545 (14.3)	196 459 (15.0)
Light smoker	434 954 (10.8)	507 523 (13.1)		154 565 (11.4)	177 693 (13.6)
Moderate smoker	226 128 (5.6)	251 170 (6.5)		74 933 (5.5)	84 914 (6.5)
Heavy smoker	115 890 (2.9)	188 857 (4.9)		38 218 (2.8)	64 107 (4.9)
Medical characteristics:					
Family history of coronary heart disease in first degree relative <60 years	481 628 (12.0)	357 987 (9.3)		164 023 (12.1)	123 039 (9.4)
Type 1 diabetes	10 060 (0.3)	11 617 (0.3)		3351 (0.2)	3932 (0.3)
Type 2 diabetes	48 022 (1.2)	58 395 (1.5)		15 872 (1.2)	19 318 (1.5)
Treated hypertension	223 494 (5.6)	164 255 (4.2)		77 694 (5.7)	56 920 (4.3)
Rheumatoid arthritis	45 700 (1.1)	20 997 (0.5)		15 139 (1.1)	7055 (0.5)
Atrial fibrillation	15 177 (0.4)	20 098 (0.5)		5229 (0.4)	6874 (0.5)
Chronic kidney disease (stage 4 or 5)	7518 (0.2)	6345 (0.2)		2583 (0.2)	2165 (0.2)
Chronic kidney disease (stage 3, 4, or 5)	19 396 (0.5)	12 254 (0.3)		6949 (0.5)	4232 (0.3)
Migraine	257 825 (6.4)	103 995 (2.7)		89 504 (6.6)	36 141 (2.8)
Corticosteroid use	96 955 (2.4)	56 533 (1.5)		31 775 (2.3)	18 634 (1.4)
HIV/AIDS	4332 (0.1)	7732 (0.2)		1595 (0.1)	2945 (0.2)
Systemic lupus erythematosus	4010 (0.1)	365 (0.0)		1349 (0.1)	134 (0.0)
Atypical antipsychotic use	19 140 (0.5)	20 123 (0.5)		6268 (0.5)	6597 (0.5)
Severe mental illness	274 069 (6.8)	167 115 (4.3)		94 724 (7.0)	57 830 (4.4)
Erectile dysfunction diagnosis or treatment	NA	90 753 (2.3)		NA	31 136 (2.4)
Erectile dysfunction diagnosis	NA	80 753 (2.1)		NA	27 727 (2.1)
Erectile dysfunction treatment	NA	28 763 (0.7)		NA	9877 (0.8)

HDL=high density lipoprotein; NA=not applicable.

Complete data for total cholesterol: HDL cholesterol ratio, body mass index, smoking, and systolic blood pressure.

*Based on standard deviation of ≥2 systolic blood pressure values.

### Predictor variables

We examined the predictor variables in box 1 based on established risk factors already included in the current version of QRISK2 and new candidate variables highlighted in the literature or National Institute for Health and Care Excellence guidelines.

**Box 1: Variables used in QRISK algorithms**

**Existing variables from QRISK2-2017**

Age at study entry (baseline)

Ethnic origin (nine categories)

Deprivation (as measured by the Townsend score, where higher values indicate higher levels of material deprivation)

Systolic blood pressure

Body mass index

Total cholesterol: high density lipoprotein cholesterol ratio

Smoking status (non-smoker, former smoker, light smoker (1-9/day), moderate smoker (10-19/day), or heavy smoker (≥20/day))

Family history of coronary heart disease in a first degree relative aged less than 60 years

Diabetes (type 1, type 2, or no diabetes)

Treated hypertension (diagnosis of hypertension and treatment with at least one antihypertensive drug)

Rheumatoid arthritis (diagnosis of rheumatoid arthritis, Felty’s syndrome, Caplan’s syndrome, adult onset Still’s disease, or inflammatory polyarthropathy not otherwise specified)

Atrial fibrillation (including atrial fibrillation, atrial flutter, and paroxysmal atrial fibrillation)

Chronic kidney disease (stage 4 or 5) and major chronic renal disease (including nephrotic syndrome, chronic glomerulonephritis, chronic pyelonephritis, renal dialysis, and renal transplant)

**New or amended risk factors considered**

Expanded definition of chronic kidney disease (to include general practitioner recorded diagnosis of chronic kidney disease stage 3 in addition to stages 4 and 5 as well as major chronic renal disease)

Measure of systolic blood pressure variability (standard deviation of repeated measures)

Diagnosis of migraine (including classic migraine, atypical migraine, abdominal migraine, cluster headaches, basilar migraine, hemiplegic migraine, and migraine with or without aura)

Corticosteroid use (*British National Formulary* (BNF) chapter 6.3.2 including oral or parenteral prednisolone, betamethasone, cortisone, depo-medrone, dexamethasone, deflazacort, efcortesol, hydrocortisone, methylprednisolone, or triamcinolone)

Systemic lupus erythematosus (including diagnosis of SLE, disseminated lupus erythematosus, or Libman-Sacks disease)

Second generation “atypical” antipsychotic use (including amisulpride, aripiprazole, clozapine, lurasidone, olanzapine, paliperidone, quetiapine, risperidone, sertindole, or zotepine)

Diagnosis of severe mental illness (including psychosis, schizophrenia, or bipolar affective disease)

Diagnosis of HIV or AIDS

Diagnosis of erectile dysfunction or treatment for erectile dysfunction (BNF chapter 7.4.5 including alprostadil, phosphodiesterase type 5 inhibitors, papaverine, or phentolamine)

From the general practice record we extracted data for demographic factors, clinical diagnoses, and clinical values. For clinical values (systolic blood pressure and body mass index) and smoking status we obtained the most recent values recorded before the baseline date. We selected the closest value to cohort entry for total cholesterol: high density lipoprotein cholesterol ratio, restricting values after the baseline date to those before the patient had a diagnosis of cardiovascular disease or was censored, and before any statin prescriptions. To assess variability in systolic blood pressure, we identified all systolic blood pressure values recorded in the five years before study entry and calculated the standard deviation where there were two or more recorded values. Use of drugs at baseline was defined as at least two prescriptions, with the most recent one no more than 28 days before the date of entry to the cohort. All other predictor variables were based on the latest information recorded in the general practice record before entry to the cohort.

### Derivation and validation of the models

We developed and validated the risk prediction algorithms using established methods^1^ ^5^ ^8^ ^10^ ^28^ and performed an initial analysis based on patients with complete variables. For our main analysis, we used multiple imputation with chained equations to replace missing values for body mass index, systolic blood pressure, standard deviation of systolic blood pressure, serum cholesterol, high density lipoprotein cholesterol, and smoking status and used these values in our main analyses.^29^ ^30^ ^31^ ^32^ We log transformed values for continuous variables that were not normally distributed for inclusion in the imputation model so that the imputed values would better match the distribution of observed values. Five imputations were carried out as this has a relatively high efficiency^33^ and was a pragmatic approach accounting for the size of the datasets and capacity of the available servers and software. In the imputation model we included all predictor variables, along with age interaction terms, the Nelson-Aalen estimator of the baseline cumulative hazard, and the outcome indicator.

Cox’s proportional hazards models were used to estimate the coefficients for each risk factor in women and men separately. We used Rubin’s rules to combine the results across the imputed datasets.^34^ Fractional polynomials^35^ were used to model non-linear risk relations with continuous variables using data from patients with recorded values to derive the fractional polynomial terms. We fitted full models initially. For consistency, we included variables from existing QRISK2 models and then retained additional variables if they had an adjusted hazard ratio of less than 0.90 or greater than 1.10 (for binary variables) and were statistically significant at the 0.01 level. We developed three main models. Model A contains the same variables as the latest version of QRISK2-2017. Model B includes the additional variables that met our inclusion criteria but not the standard deviation of serial systolic blood pressure values. Model C is the same as model B except that it includes the standard deviation of serial systolic blood pressure values. We examined interactions between new predictor variables and age at study entry and included significant interactions in models B and C along with interactions already included in QRISK2.

From the final models we used the regression coefficients for each variable as weights, which we combined with the baseline survivor function evaluated up to 15 years to derive risk equations over a period of 15 years of follow-up.^36^ This enabled us to derive risk estimates for each year of follow-up, with a specific focus on 10 year risk estimates. We estimated the baseline survivor function based on zero values of centred continuous variables, with all binary predictor values set to zero.

### Validation of the models

In the validation cohort we used multiple imputation to replace missing values for body mass index, systolic blood pressure, standard deviation of systolic blood pressure, serum cholesterol, high density lipoprotein cholesterol, and smoking status. We carried out five imputations. The risk equations for women and men obtained from the derivation cohort for models A, B, and C were applied to the validation cohort and measures of discrimination calculated. As in previous studies,^4^ we calculated R^2^ values (explained variation where higher values indicate a greater proportion of variation in time to cardiovascular disease diagnosis is explained by the model 37), D statistic^38^ (a measure of discrimination where higher values indicate better discrimination), and Harrell’s C statistic at 10 years and combined these across datasets using Rubin’s rules. Harrell’s C statistic^39^ is a measure of discrimination that is similar to the area under a receiver operating characteristic curve but takes account of the censored nature of the data.

We assessed calibration (comparing the mean predicted risks at 10 years with the observed risk by 10th of predicted risk). The observed risks were obtained using the Kaplan-Meier estimates evaluated at 10 years. We also evaluated performance in each age group (<40, 40-59, ≥60 years), ethnic origin subgroup, and each comorbidity and treatment subgroup. Performance was also evaluated by calculating Harrell’s C statistics in individual general practices and combining the results using meta-analytical techniques for comparison with a previous study of QRISK2.^9^

### Reclassification statistics

In line with current NICE guidelines,^20^ we classified patients as being at high risk of cardiovascular disease if their 10 year risk was 10% or greater. We compared predicted risks for our final models (QRISK3) with the latest version of QRISK2-2017 to determine the percentage of patients who would be reclassified at this threshold according to each model. Among the reclassified patients we also calculated the observed risks of cardiovascular disease at 10 years using the Kaplan-Meier method.

To maximise the power and generalisability of the results we used all the relevant patients on the database. STATA (version 14) was used for all analyses. The study adhered to the TRIPOD (Transparent Reporting of a multivariable prediction model for Individual Prognosis Or Diagnosis) statement for reporting.^40^

### Patient involvement

Over the past 10 years since the original publication of QRISK^1^ there has been extensive discussion about methods for assessment of cardiovascular risk. This has included a series of public stakeholder consultations in relation to updates of NICE guidance on lipid modification,^20^ the NHS Quality and Outcomes Framework, and NHS Health Check.^21^ We therefore decided to focus on issues highlighted in NICE guidance and the literature rather than to consult patient or professional groups. We decided it would be more transparent and effective to discuss the addition of new variables once the paper was published and the relative contribution of individual risk factors had been quantified. Given the widespread implementation of QRISK2 across the NHS and its inclusion in guidelines, this would give time for feedback from a range of stakeholders (including patient groups and charities) as to which changes would be most beneficial and how improvements might be implemented. 

## Results

### Study population

Overall, 1309 practices contributing to the QResearch database in England met our inclusion criteria. Of these, 981 were randomly assigned to the derivation dataset and the remainder (n=328) to a validation cohort. For the derivation cohort we identified 8 602 833 patients aged 25-84 years. We excluded 31 433 (0.4%) with no recorded Townsend score, 344 669 (4.0%) with a diagnosis of cardiovascular disease at baseline recorded on the general practice or Hospital Episode Statistics record, and 336 928 (3.9%) prescribed statins at baseline. Overall, 7 889 803 patients were included in the derivation analysis.

For the validation cohort we identified 2 918 082 patients aged 25-84 years. We excluded 13 862 (0.5%) with no recorded Townsend score, 118 057 (4.0%) with a diagnosis of cardiovascular disease recorded on the general practice or Hospital Episode Statistics record, and 114 865 (3.9%) prescribed statins at baseline. In total, 2 671 298 patients were included in the validation analysis.

### Baseline characteristics

Table 1 shows the baseline characteristics of men and women in the derivation and validation cohorts. In the derivation cohort, self assigned ethnic origin was recorded for 64.9% of women and 59.7% of men, smoking status for 85.0% and 77.7%, respectively, systolic blood pressure for 82.8% and 68.3%, respectively, body mass index for 72.8% and 64.0%, respectively, and total cholesterol: high density lipoprotein cholesterol ratio for 39.8% and 37.9%, respectively. Complete information for smoking status, systolic blood pressure, body mass index, and total cholesterol: high density lipoprotein cholesterol ratio was provided for 28.5% of women and 24.6% of men. At least two systolic blood pressures were recorded for 77.7% of women and 64.0% of men from which the standard deviations were calculated. These values were similar to corresponding values for both sexes in the validation cohort (table 1).

Table 1 also shows comorbidities at study entry. For the new variables of interest, severe mental illness was recorded for 6.8% of women and 4.3% of men, migraine for 6.4% and 2.7%, respectively, chronic kidney disease (stage 3, 4, or 5) for 0.5% and 0.3%, respectively; prescribed atypical antipsychotics for 0.5% of women and men, and prescribed corticosteroids for 2.4% and 1.5%, respectively, and 2.3% of men had a diagnosis of or treatment for erectile dysfunction. SLE was recorded for 0.1% of women and less than 0.1% of men and HIV/AIDS for 0.1% of women and 0.2% of men. The mean of the most recent systolic blood pressure values was 123.2 mm Hg in women and 129.2 mm Hg in men and the mean of the standard deviations of repeated systolic blood pressure values was 9.3 in women and 9.9 in men.

### Incidence rates of cardiovascular disease

Table 2 shows the numbers of patients with a new diagnosis of cardiovascular disease during follow-up by age group (five year intervals) in women and men in the derivation cohort based on the linked general practice, hospital, and Office for National Statistics morality records. In the derivation cohort, we identified 363 565 incident cases of cardiovascular disease arising from 50.8 million person years of observation. The incidence of cardiovascular disease increased steeply by age group and values were higher in men than women for all age groups. Table 2 in the web appendix shows a similar breakdown by nine ethnic groups. For example, 4758 events occurred in Indian women and men arising from 8 819 177 person years of observation and 417 events in Chinese women and men arising from 210 267 person years of observation.

**Table 2 tbl2:** Incidence rates of cardiovascular disease per 1000 person years in derivation cohort

Age group (years)	Women				Men		
	Incident cases	Person years	Rate per 1000 person years (95% CI)		Incident cases	Person years	Rate per 1000 person years (95% CI)
25-29	832	3 455 662	2.4 (2.2 to 2.6)		1351	3 379 716	4 (3.8 to 4.2)
30-34	1878	3 802 577	4.9 (4.7 to 5.2)		3823	3 880 890	9.9 (9.5 to 10.2)
35-39	3636	3 551 460	10.2 (9.9 to 10.6)		7963	3 748 285	21.2 (20.8 to 21.7)
40-44	5651	2 971 995	19 (18.5 to 19.5)		12 750	3 192 048	39.9 (39.3 to 40.6)
45-49	8272	2 581 104	32 (31.4 to 32.7)		17 763	2 672 642	66.5 (65.5 to 67.4)
50-54	12 022	2 490 263	48.3 (47.4 to 49.1)		24 040	2 437 106	98.6 (97.4 to 99.9)
55-59	14 524	1 944 140	74.7 (73.5 to 75.9)		25 464	1 796 342	141.8 (140.0 to 143.5)
60-64	18 471	1 625 795	113.6 (112.0 to 115.3)		27 021	1 372 104	196.9 (194.6 to 199.3)
65-69	22 510	1 314 303	171.3 (169.0 to 173.5)		26 903	1 013 291	265.5 (262.3 to 268.7)
70-74	25 462	1 015 263	250.8 (247.7 to 253.9)		24 549	691 866	354.8 (350.4 to 359.3)
75-79	26 883	765 681	351.1 (346.9 to 355.3)		19 820	438 861	451.6 (445.4 to 458.0)
80-84	20 408	424 994	480.2 (473.7 to 486.8)		11 569	198 481	582.9 (572.4 to 593.6)
Total	160 549	25 943 236	61.9 (61.6 to 62.2)		203 016	24 821 632	81.8 (81.4 to 82.1)

Table 3 in the web appendix shows the source of the data that first identified the incident event by type of event in the derivation cohort. It also shows the number and percentage of cases that were identified only using general practice data with no subsequent evidence of cardiovascular disease on hospital or mortality records. Of the 363 565 incident events, 78 327 (21.5%) were myocardial infarction, 152 141 (41.8%) were angina, 49 504 (13.6%) were transient ischaemic attack, and 83 593 (23.0%) were ischaemic strokes. Overall, 92 936 (25.6% of all 363 565 events) were only recorded on the general practice record, with the most common condition being transient ischaemic attack (27 227 events).

**Table 3 tbl3:** Adjusted hazard ratios (95% confidence interval) for cardiovascular disease in women in the derivation cohort

Predictor variables	Model A*	Model B†	Model C‡
Townsend score (per 5 unit increase)§	1.48 (1.46 to 1.51)	1.47 (1.45 to 1.50)	1.47 (1.45 to 1.50)
Ethnic origin:			
White or not recorded	1.00	1.00	1.00
Indian	1.32 (1.26 to 1.38)	1.32 (1.26 to 1.39)	1.32 (1.26 to 1.39)
Pakistani	1.76 (1.66 to 1.87)	1.76 (1.66 to 1.87)	1.76 (1.66 to 1.86)
Bangladeshi	1.33 (1.23 to 1.44)	1.35 (1.25 to 1.46)	1.34 (1.24 to 1.45)
Other Asian	1.07 (0.985 to 1.16)	1.08 (0.995 to 1.17)	1.08 (0.992 to 1.17)
Black Caribbean	0.836 (0.791 to 0.884)	0.844 (0.798 to 0.892)	0.843 (0.797 to 0.891)
Black African	0.660 (0.605 to 0.721)	0.677 (0.620 to 0.740)	0.675 (0.618 to 0.737)
Chinese	0.710 (0.612 to 0.823)	0.726 (0.625 to 0.842)	0.722 (0.622 to 0.837)
Other	0.836 (0.786 to 0.890)	0.843 (0.792 to 0.897)	0.843 (0.791 to 0.897)
Smoking status§:			
Non-smoker	1.00	1.00	1.00
Former smoker	1.16 (1.12 to 1.19)	1.14 (1.11 to 1.18)	1.14 (1.11 to 1.18)
Light smoker	1.79 (1.73 to 1.85)	1.76 (1.70 to 1.82)	1.75 (1.70 to 1.81)
Moderate smoker	1.98 (1.91 to 2.05)	1.95 (1.88 to 2.02)	1.95 (1.88 to 2.02)
Heavy smoker	2.39 (2.30 to 2.49)	2.34 (2.25 to 2.44)	2.34 (2.25 to 2.43)
Medical characteristics:			
Family history of coronary heart disease in first degree relative <60 years§	1.59 (1.56 to 1.63)	1.58 (1.54 to 1.61)	1.58 (1.54 to 1.61)
Type 1 diabetes§	5.66 (5.11 to 6.26)	5.66 (5.12 to 6.26)	5.62 (5.08 to 6.22)
Type 2 diabetes§	2.95 (2.76 to 3.15)	2.92 (2.73 to 3.13)	2.91 (2.72 to 3.11)
Treated hypertension§	1.75 (1.68 to 1.82)	1.71 (1.64 to 1.78)	1.66 (1.60 to 1.73)
Rheumatoid arthritis	1.32 (1.28 to 1.36)	1.24 (1.21 to 1.28)	1.24 (1.20 to 1.27)
Atrial fibrillation§	5.09 (4.35 to 5.95)	4.94 (4.23 to 5.78)	4.92 (4.20 to 5.75)
Chronic kidney disease (stage 4 or 5)§	2.31 (2.02 to 2.65)	NA	NA
Chronic kidney disease (stage 3, 4, or 5)§	NA	1.94 (1.72 to 2.19)	1.92 (1.70 to 2.17)
Migraine§	NA	1.36 (1.31 to 1.41)	1.35 (1.30 to 1.40)
Corticosteroid use§	NA	1.82 (1.74 to 1.90)	1.81 (1.74 to 1.89)
Systemic lupus erythematosus§	NA	2.15 (1.79 to 2.57)	2.14 (1.78 to 2.56)
Atypical antipsychotic use	NA	1.29 (1.21 to 1.38)	1.29 (1.21 to 1.37)
Severe mental illness	NA	1.14 (1.11 to 1.16)	1.13 (1.11 to 1.16)
Total cholesterol: HDL cholesterol ratio (per unit increase)	1.17 (1.16 to 1.17)	1.17 (1.16 to 1.17)	1.17 (1.16 to 1.17)
Systolic blood pressure (per 20 unit increase)	1.14 (1.14 to 1.15)	1.15 (1.14 to 1.15)	1.14 (1.13 to 1.15)
Standard deviation of blood pressure (per 10 unit increase)	NA	NA	1.08 (1.07 to 1.09)

NA=not applicable; HDL=high density lipoprotein.

*Includes chronic kidney disease (stage 4 or 5) fractional polynomial terms for age (age and age^−2^) and body mass index (BMI^−2^ and BMI^−2^ln(BMI)), and interactions with age for body mass index, systolic blood pressure, Townsend score, family history of coronary heart disease, treated hypertension, atrial fibrillation, type 1 diabetes, type 2 diabetes, chronic kidney disease, and smoking status.

†Same as model A with chronic kidney disease (stage 3, 4, or 5), extra variables listed in table, and additional age interactions for: migraine, corticosteroid use, and systemic lupus erythematosus.

‡Same as model B but with standard deviation of systolic blood pressure.

§Interaction with age; hazard ratios evaluated at mean age.

The median follow-up in the derivation cohort was 4.4 years (interquartile range 1.6-10.8) and 2 141 841 patients had 10 years or more of follow-up and 1 090 704 had 15 years or more of follow-up. Of the 7 889 803 patients in the derivation cohort, 696 387 (8.8%) started using statins after entry to the cohort and before having a new diagnosis of cardiovascular disease or being censored. Of the 50 764 868 person years of follow-up, 46 940 777 person years were free from statin use (92.5%).

In the validation cohort, the median follow up was 4.4 years (interquartile range 1.6-10.8) and 728 704 patients had 10 years or more of follow-up and 380 387 had 15 years or more of follow-up. 

### Predictor variables

Table 3 shows the adjusted hazard ratios for women in the derivation cohort and table 4 shows the corresponding values for men. Of the new risk factors, all met our model inclusion criteria except for HIV/AIDS, which was associated with a 25% increased risk in women and 17% increased risk in men, but these were not statistically significant at the 0.01 level. Model A is the latest version of QRISK2 (2017). Model B includes the additional variables that met our inclusion criteria. Model C is the same as model B except that it includes the standard deviation of serial systolic blood pressure values.

**Table 4 tbl4:** Adjusted hazard ratios (95% confidence interval) for cardiovascular disease in men in the derivation cohort

Predictor variables	Model A*	Model B†	Model C‡
Townsend score (per 5 unit increase)§	1.19 (1.17 to 1.20)	1.18 (1.17 to 1.20)	1.18 (1.17 to 1.20)
Ethnic origin:			
White or not recorded	1.00	1.00	1.00
Indian	1.31 (1.26 to 1.36)	1.32 (1.27 to 1.37)	1.32 (1.27 to 1.37)
Pakistani	1.62 (1.54 to 1.69)	1.61 (1.53 to 1.68)	1.61 (1.53 to 1.68)
Bangladeshi	1.70 (1.61 to 1.79)	1.70 (1.62 to 1.80)	1.70 (1.61 to 1.79)
Other Asian	1.03 (0.968 to 1.10)	1.04 (0.970 to 1.11)	1.04 (0.970 to 1.11)
Black Caribbean	0.700 (0.663 to 0.738)	0.700 (0.663 to 0.739)	0.699 (0.662 to 0.738)
Black African	0.671 (0.623 to 0.722)	0.672 (0.625 to 0.724)	0.670 (0.623 to 0.721)
Chinese	0.652 (0.574 to 0.740)	0.66 (0.581 to 0.749)	0.660 (0.582 to 0.749)
Other	0.770 (0.729 to 0.814)	0.77 (0.729 to 0.813)	0.769 (0.728 to 0.812)
Smoking status§:			
Non-smoker	1.00	1.00	1.00
Former smoker	1.22 (1.19 to 1.25)	1.21 (1.18 to 1.24)	1.21 (1.18 to 1.24)
Light smoker	1.75 (1.71 to 1.79)	1.74 (1.70 to 1.78)	1.74 (1.70 to 1.78)
Moderate smoker	1.91 (1.86 to 1.96)	1.90 (1.85 to 1.95)	1.89 (1.84 to 1.94)
Heavy smoker	2.22 (2.16 to 2.29)	2.21 (2.14 to 2.28)	2.20 (2.14 to 2.27)
Medical characteristics:			
Family history of coronary heart disease in first degree relative <60 years§	1.73 (1.7 to 1.76)	1.72 (1.69 to 1.75)	1.72 (1.69 to 1.75)
Type 1 diabetes§	3.59 (3.31 to 3.90)	3.47 (3.20 to 3.77)	3.44 (3.17 to 3.73)
Type 2 diabetes§	2.42 (2.29 to 2.57)	2.37 (2.24 to 2.51)	2.36 (2.23 to 2.50)
Treated hypertension§	1.76 (1.69 to 1.83)	1.73 (1.67 to 1.80)	1.68 (1.61 to 1.74)
Rheumatoid arthritis	1.30 (1.25 to 1.35)	1.24 (1.19 to 1.28)	1.23 (1.19 to 1.28)
Atrial fibrillation§	2.46 (2.18 to 2.78)	2.44 (2.16 to 2.76)	2.42 (2.14 to 2.73)
Chronic kidney disease (stage 4 or 5)§	2.39 (2.13 to 2.68)	NA	NA
Chronic kidney disease (stage 3, 4, or 5)§	NA	2.09 (1.87 to 2.34)	2.05 (1.83 to 2.29)
Migraine§	NA	1.29 (1.24 to 1.35)	1.29 (1.24 to 1.34)
Corticosteroid use§	NA	1.58 (1.51 to 1.66)	1.58 (1.5 to 1.66)
Systemic lupus erythematosus	NA	1.55 (1.15 to 2.10)	1.55 (1.15 to 2.10)
Atypical antipsychotic use	NA	1.15 (1.07 to 1.23)	1.14 (1.06 to 1.22)
Severe mental illness	NA	1.13 (1.11 to 1.16)	1.13 (1.10 to 1.15)
Erectile dysfunction or treatment§	NA	1.25 (1.18 to 1.33)	1.25 (1.18 to 1.33)
Total cholesterol: HDL cholesterol ratio (per unit increase)	1.19 (1.18 to 1.19)	1.19 (1.18 to 1.19)	1.19 (1.18 to 1.19)
Systolic blood pressure (per 20 unit increase)	1.14 (1.13 to 1.15)	1.14 (1.14 to 1.15)	1.14 (1.13 to 1.14)
Standard deviation of blood pressure (per 10 unit increase)	NA	NA	1.11 (1.09 to 1.12)

NA=not applicable; HDL=high density lipoprotein.

Includes chronic kidney disease (stage 4 or 5) fractional polynomial terms for age (age^-1^ and age^3^) and body mass index (BMI^−2^ and BMI^−2^ln(BMI)), and interactions with age for body mass index, systolic blood pressure, Townsend score, family history of coronary heart disease, treated hypertension, atrial fibrillation, type 1 diabetes, type 2 diabetes, chronic kidney disease, and smoking status.

†Same as model A with chronic kidney disease (stage 3, 4, or 5), extra variables listed in table, and additional age interactions for: migraine, corticosteroid use, and erectile dysfunction.

‡Same as model B but with standard deviation of systolic blood pressure.

§Interaction with age; hazard ratios evaluated at mean age.

The supplementary figure shows graphs of the adjusted hazard ratios for model B for the fractional polynomial terms for age and body mass index as well as the interaction terms between age and relevant predictor variables, as listed in the footnotes of tables 3 and 4[Table tbl3 tbl4]. For the new variables of interest in model B, migraine was associated with a 36% increased risk of cardiovascular disease for women and a 29% increased risk for men, corticosteroids were associated with an 82% increased risk for women and 58% increased risk for men, SLE was associated with a 115% increased risk for women and a 55% increased risk for men, atypical antipsychotics were associated with a 29% increased risk for women and a 15% increased risk for men, severe mental illness was associated with a 14% increased risk for women and a 13% increased risk for men. Erectile dysfunction was associated with a 25% increased risk. Where there were age interactions these values relate to risks evaluated at the mean ages. The full list of age interactions is shown in the footnotes for tables 3 and 4[Table tbl3 tbl4]. For the new variables, there were statistically significant interactions between age and migraine as well as age and corticosteroid use in both sexes. In women, there was also a statistically significant interaction between age and SLE. In men, there was also a statistically significant interaction between age and erectile dysfunction. For each of these interactions, hazard ratios for the predictors were higher at younger ages compared with older ages, except for erectile dysfunction in men, where hazard ratios were highest for men aged around age 45 and then declined gradually with increasing age.

For model C, the standard deviation of systolic blood pressure values was included in the model in addition to the single most recent systolic blood pressure value. Overall a 10 unit increase in the standard deviation of systolic blood pressure was associated with an 8% increased risk of cardiovascular disease in women (table 3) and an 11% increased risk in men (table 4).

Tables 4 and 5[Table tbl4 tbl5] in the web appendix show the results of complete case analyses for models B and C for women and men, respectively (ie, the results based on patients with complete data). The hazard ratios associated with total cholesterol: high density lipoprotein cholesterol ratio, systolic blood pressure, and standard deviation of systolic blood pressure were similar to those obtained in the main models using multiply imputed data.

**Table 5 tbl5:** Mean (95% confidence interval) performance of models A, B, and C in the validation cohort in women and men aged 25-84 years

Statistic	Model A			Model B			Model C	
	Women	Men		Women	Men		Women	Men
D statistic*	2.48 (2.46 to 2.50)	2.25 (2.24 to 2.27)		2.48 (2.46 to 2.50)	2.26 (2.24 to 2.27)		2.49 (2.47 to 2.51)	2.26 (2.25 to 2.28)
Harrell's C*	0.879 (0.878 to 0.880)	0.858 (0.856 to 0.859)		0.880 (0.878 to 0.881)	0.858 (0.857 to 0.859)		0.880 (0.879 to 0.882)	0.858 (0.857 to 0.860)
R^2^ (%)†	59.6 (59.2 to 60.0)	54.8 (54.4 to 55.1)		59.5 (59.2 to 59.9)	54.8 (54.5 to 55.2)		59.6 (59.3 to 60.0)	55.0 (54.6 to 55.3)

*A measure of discrimination. Higher values indicate better discrimination.

†Measures explained variation in time to diagnosis. Higher values indicate more variation is explained.

### Validation

#### Discrimination

Table 5 shows the performance of each algorithm in the validation cohort for women and men for each of models A, B, and C. For model B in women, the algorithm explained 59.5% of the variation in time to diagnosis of cardiovascular disease (R^2^), the D statistic was 2.48, and the Harrell’s C statistic was 0.88. The corresponding values for men were 54.8%, 2.26, and 0.86. Measures of performance were similar for all three models.

Table 6 in the web appendix shows the validation statistics for model B in various subgroups, including three age groups, ethnic groups, and in those with specific comorbidities. The highest performance values by ethnic origin were in Chinese women (R^2^=64.7%; D=2.77; Harrell’s C=0.91) and the lowest values were in Caribbean women (R^2^=51.6%; D=2.11; Harrell’s C=0.85). Performance values were highest in the youngest age group (25-39 years) and lowest in the oldest age group (60-84 years).

**Table 6 tbl6:** Clinical examples update with new models

Characteristics	Examples					
	1	2	3	4	5	6
Sex	Male	Male	Male	Female	Female	Male
Age (years)	44	45	48	55	61	48
Body mass index	27.2	22.4	29.7	24.9	33.7	30
Total cholesterol: HDL cholesterol ratio	6.1	6.3	5	3.2	4.8	4.2
Systolic blood pressure	130	115	124	130	155	140
Ethnic origin	White	White	White	White	Black African	White
Smoking status	Heavy smoker	Non-smoker	Light smoker	Moderate smoker	Former smoker	Non-smoker
Family history of coronary heart disease	No	No	No	Yes	No	No
Type 1 diabetes	No	No	No	No	No	No
Type 2 diabetes	No	Yes	No	No	No	No
Treated hypertension	No	No	No	No	No	Yes
Rheumatoid arthritis	No	No	No	No	No	No
Atrial fibrillation	No	No	No	No	No	No
Chronic kidney disease (stage 3, 4, or 5)	No	No	No	No	No	No
Migraine	Yes	No	Yes	No	No	Yes
Corticosteroid use	No	No	Yes	No	No	No
Systemic lupus erythematosus	No	No	No	No	No	No
Atypical antipsychotic use	No	No	No	No	Yes	No
Severe mental illness	No	No	No	No	Yes	No
Erectile dysfunction or treatment	No	Yes	No	NA	NA	No
Standard deviation of systolic blood pressure	6	40	3.1	22	33	No
Model A 10 year predicted risk	9.2	8.3	6.4	11	9.4	9.2
Model B 10 year predicted risk	11	9.9	11	10	13	11
Model C 10 year predicted risk	11	13	11	11	15	9.5

NA=not applicable HDL=high density lipoprotein.

For the subgroup of women with type 1 diabetes the R^2^ was 47.3%, D statistic was 1.94, and Harrell’s C statistic was 0.82. The corresponding values for men with type 1 diabetes were 45.6%, 1.87, and 0.80. For the subgroup of women with type 2 diabetes the R^2^ was 25.2%, D statistic was 1.19, and Harrell’s C statistic was 0.70. The corresponding values for men with type 2 diabetes were 22.9%, 1.12, and 0.70.

Figure 1 shows the funnel plots of Harrell’s C statistic for model B across the 328 practices in the validation cohort. The funnel plots show Harrell’s C statistic for each general practice versus the number of cardiovascular events in each practice in women and men separately. Practices with fewer cardiovascular events had wider variation in the C statistic than practices with more events. The summary (average) C statistic for women was 0.874 (95% confidence interval 0.869 to 0.880) from a random effects meta-analysis. The I^2^ value (ie, the percentage of total variation in C statistics owing to between practice heterogeneity) was 93.3%. The approximate 95% prediction interval for the true C statistic in women in a new practice was 0.79 to 0.96. The summary C statistic for men was 0.851 (95% confidence interval, 0.847 to 0.855) from a random effects meta-analysis. The I^2^ value was 84.2%. The approximate 95% prediction interval for the true C statistic in men in a new practice was 0.79 to 0.91.

**Figure f1:**
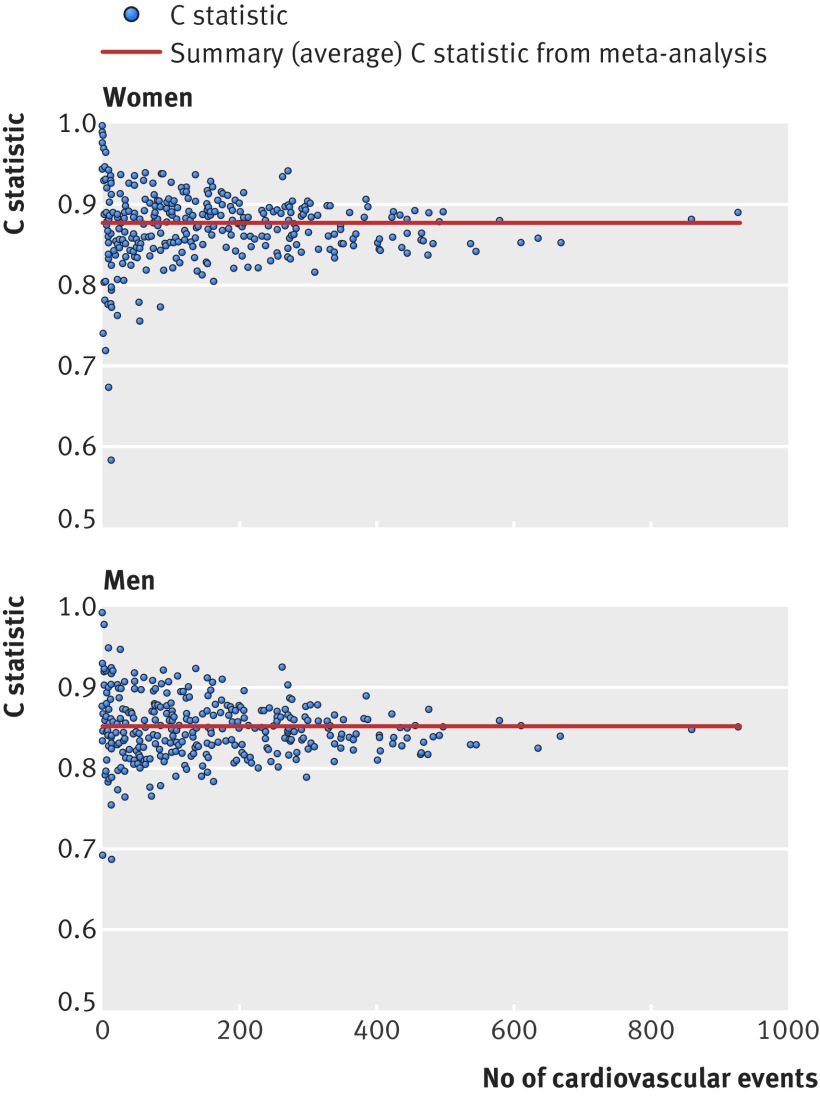
**Fig 1** Funnel plots of discrimination performance (Harrell’s C statistic) across 328 general practices

#### Calibration

In women, the mean 10 year predicted risk was 4.7% for models A, B, and C. The observed 10 year risk was 5.8% (95% confidence interval 5.8% to 5.9%). In men, the mean 10 year predicted risk was 6.4% for models A, B, and C. The observed 10 year risk was 7.5% (7.5% to 7.6%). Figure 2 shows the mean predicted risks and observed risks at 10 years by 10th of predicted risk, applying each algorithm to all women and men in the validation cohort and to separate age groups (25-39, 40-59, and 60-84 years). There was close correspondence between the mean predicted risks and the observed risks within each model 10th overall and in each age group in women and men indicating that the algorithms were well calibrated. The exception was in those aged 25-39 where mean predicted risks were slightly higher than observed risks.

**Figure f2:**
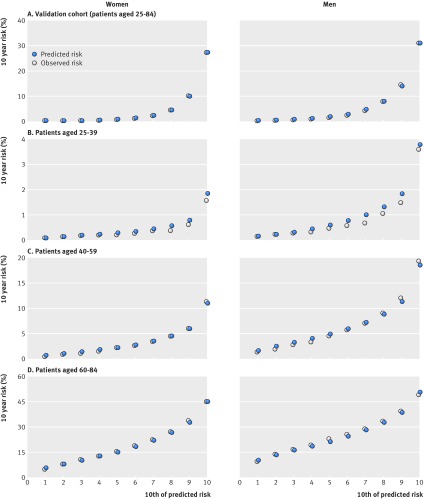
**Fig 2** Predicted and observed 10 year cardiovascular disease risk by 10th of predicted risk

### Reclassification

Overall, there were 2 671 298 patients in the validation cohort. Of these, 458 263 (17.2%) had a 10 year risk score of 10% or greater using model A; 458 869 (17.2%) using model B, and 458 868 (17.2%) using model C.

Using model A, the number of patients with a 10 year risk score of 15% or more was 308 130 (11.5%) and with a risk of 20% of more was 214 451 (8.0%). The corresponding numbers for models B and C were similar.

Of 458 263 patients with a 10 year predicted risk score of 10% or more using model A, 10 948 (2.4%) would be reclassified as low risk (predicted risk <10% over 10 years) using model B. The 10 year observed risk among these reclassified patients was 10.3% (95% confidence interval 9.6% to 11.1%), just above the 10% threshold. Conversely, of the 2 213 035 classified as low risk (predicted risk <10% over 10 years) using model A, 11 554 (0.5%) would be reclassified as high risk using model B. The 10 year observed risk among these reclassified patients was 12.2% (11.4% to 13.1%), above the 10% threshold.

Of the 458 869 patients with a 10 year predicted risk score of 10% or more using model B, 9102 (2.0%) would be reclassified as low risk using model C. The 10 year observed risk among these reclassified patients was 9.6% (95% confidence interval 8.9% to 10.5%), marginally below the 10% threshold. Conversely, of the 2  213 429 with a 10 year predicted risk score of less than 10% using model B, 9101 (2.4%) would be reclassified as high risk using model C. The 10 year observed risk among these reclassified patients was 10.7% (9.9% to 11.6%), marginally above the 10% threshold.

### Clinical examples

Table 6 shows clinical examples where use of model A, B, or C would result in a reclassification above or below the 10% threshold. Figures 3 and 4 show screenshots of the updated web calculator with a clinical example which can be found at www.qrisk.org.

**Figure f3:**
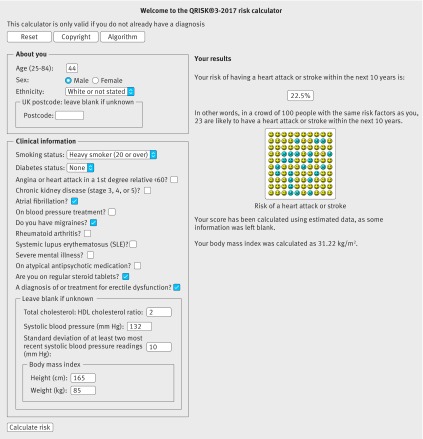
**Fig 3** 10 year risk of 22.5% based on model C for a white man, aged 44, heavy smoker, total cholesterol: high density lipoprotein (HDL) cholesterol ratio of 2, systolic blood pressure of 132 mm Hg, standard deviation of systolic blood pressure of 10 mm Hg, body mass index of 31.22, atrial fibrillation, erectile dysfunction, migraine, and steroid use

**Figure f4:**
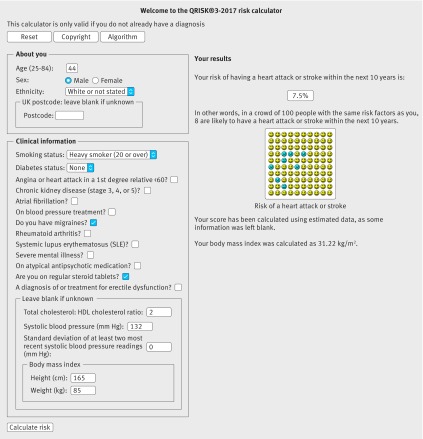
**Fig 4** 10 year risk ratio of 7.5% based on model C for white man, aged 44, heavy smoker, total cholesterol: high density lipoprotein cholesterol ratio of 2, systolic blood pressure of 132 mm Hg, standard deviation of systolic blood pressure of 0, body mass index of 31.22, migraine, steroid use, no atrial fibrillation, and no erectile dysfunction

## Discussion

We have developed and validated updated algorithms (QRISK3) to predict 10 year risk of cardiovascular disease in women and men aged 25-84 years. The algorithms incorporate established predictor variables from QRISK2 as well as new variables associated with increased risk of cardiovascular disease. These include an expanded definition of chronic kidney disease to include chronic kidney disease stage 3, migraine, corticosteroid use, systemic lupus erythematosus (SLE), atypical antipsychotic use, severe mental illness, erectile dysfunction, and a measure of blood pressure variability (standard deviation of repeated values). We have produced three main final models: model A, which includes the same variables and coefficients as the current version of QRISK2-2017; model B, which includes the new variables and the latest systolic blood pressure value and is for use where only the current reading is available; and our preferred model C, which additionally includes a measure of blood pressure variability that may be more suitable for integration into general practice computer systems where longitudinal repeated values are likely to be available. Although in population terms the overall performance of all three models is similar, for those who have one or more of the conditions included in the newer models, having the additional risk taken into account could result in the difference between taking or not taking risk reducing treatment. The increased complexity is unlikely to affect the take-up of the new models as they are designed to be calculated automatically from the electronic patient record.

### Comparisons with the literature

The hazard ratios of the new risk variables included in our final models are similar in both magnitude and direction to those reported in other studies.^25^

### Migraine

Sufficient pathophysiological and epidemiological evidence have now accumulated for some experts to propose that migraine should be included as a marker for future cardiovascular disease.^41^ Our results support this since we found that migraine was associated with a 36% increased risk of cardiovascular disease for women and 29% for men (model B). This is consistent with the increased risk of 42% in 27 840 women aged 45 and over in the Women’s Health Study^42^ and the increased risk of 50% reported in a recent study of 115 541 women aged 25-42 recruited to the Nurses’ Health Study II.^25^ In our study, migraine was recorded in 6.4% of women and 2.7% of men. This is less than the 16% reported in the Nurse’s Health Study II^25^ and the 18.4% in the Women’s Health Study^42^ and might reflect differences in cohort selection, clinical setting, consulting patterns, diagnostic criteria, or recording of diagnoses. For example, our study is based on routinely collected health records and uses diagnoses recorded by clinicians before entry to the cohort. In contrast, the Nurses’ Health Study II used self report questionnaires at three time points over a six year period. Our study, which also includes men, is much larger than previous studies.^25^ ^42^ While our study may be more representative of the general population than patients recruited to a trial, it is also susceptible to ascertainment bias. This would be the case if not all patients with migraine visited their general practitioner and not all of those diagnoses are recorded. Conversely, the Nurses’ Health Study II and the Women’s Health Study may be subject to recall bias owing to the use of self reported questionnaires inquiring about historical diagnoses. Also, our definition of migraine included a range of subtypes so it is not possible to say which of these are associated with the additional risk of being categorised as having migraine. For example, the bulk of the risk could be coming from those with migraine with aura rather than other subtypes.^43^ While the magnitude of the increased risk associated with migraine is relatively small at the individual level, it is important at the population level since migraine is so prevalent.^41^ Hence there is good justification for including clinician recorded diagnosed migraine in our new models.

### Corticosteroids and antipsychotics

The National Institute for Health and Care Excellence guidance states that cardiovascular disease risk scores will underestimate cardiovascular risk among people who are taking medicines that cause dyslipidaemia such as antipsychotic drugs or corticosteroids.^20^ In line with other studies,^44^ we found evidence to support the increased risk with corticosteroids despite simultaneous adjustment of lipid levels. Current corticosteroids (defined as ≥2 prescriptions, with the most recent one within the 28 days before study entry) were prescribed for 2.4% of women and 1.5% of men and were associated with an 82% increased cardiovascular risk in women and 58% increased risk in men. This is similar to the increased risks with corticosteroids found in other studies.^45^ ^46^ However, our definition was relatively simple (and could be used in clinical practice) but did not account for duration of use and dose and so allows for substantial heterogeneity in the indications for steroid use, and the effect may not apply equally to those with different levels of exposure. Similarly, atypical antipsychotic drugs were prescribed to 0.5% of men and women and were associated with a 29% increased cardiovascular risk in women and 15% increased risk in men. Both corticosteroids and atypical antipsychotics therefore seem to be clinically important variables to include in QRISK, taking account of the magnitude of the risk and the potential numbers of patients affected.

### Severe mental illness

The NICE guidance highlights the increased cardiovascular risk associated with severe mental illness,^20^ although this is contrary to a recent systematic review and meta-analysis, which failed to find sufficient evidence to support this conclusion.^47^ Our study found that 6.8% of women and 4.3% of men had a diagnosis of severe mental illness affects and it was associated with a 14% increased risk of cardiovascular disease for women and a 13% increased risk for men (model B). This is independent of the risk associated with atypical antipsychotics and hence both factors have been included separately as they will have a compound effect on cardiovascular risk. Clinicians will now be able to provide better information to these patients both about interventions to reduce cardiovascular risk and about the potential effects of atypical antipsychotics.

### SLE

The NICE guidance on lipid modification^20^ highlights the increased cardiovascular risk associated with SLE. The excess risk is thought to be driven largely by inflammation and an active immunological response.^48^

Reduction in risk in patients with SLE may need both modification of SLE specific factors such as disease activity and drug therapy as well as modification of traditional cardiovascular disease risk factors, although the role of anti-inflammatory treatments is not yet clear.^48^ We found that a diagnosis of SLE is associated with a 115% increased risk for women and a 55% increased risk for men. While SLE is relatively uncommon (affecting 0.1% of women and rarely affecting men), the magnitude of the increased risk is high (substantially higher than rheumatoid arthritis for example) particularly at younger ages (hazard ratios were >2 for ages ≤45 years). This makes it an important risk factor for these patients and is consistent with other studies examining cardiovascular outcomes in patients with these conditions.^48^

### Chronic kidney disease

The NICE guidance^20^ states “do not use a risk assessment tool in people with an estimated glomerular filtration rate (eGFR) of less than 60/mL/1.73 m^2^ and/or albuminuria. These people are at increased risk of cardiovascular disease . . . Atorvastatin should be offered to people with CKD [chronic kidney disease].” Our expanded definition of chronic kidney disease now includes chronic kidney disease stage 3 (eGFR 30-59/mL/1.73 m^2^) in addition to stages 4 and 5, in line with other published studies.^49^ This means QRISK3 can be used in such patients and will provide them with better information to inform their choice about use of statins and potentially other non-drug interventions to reduce their cardiovascular risk and to “encourage the person to participate in reducing their risk” in line with the recommendations for other patients.

### Type 1 diabetes

Although the NICE guidance on lipid modification^20^ recommends the use of QRISK2 in patients with type 2 diabetes, it states “do not use a risk assessment tool to assess CVD [cardiovascular disease] risk in patients with type 1 diabetes.” Instead it recommends that “statin treatment is offered to all patients with type 1 diabetes who are older than 40 years or have had diabetes for more than 10 years or have established nephropathy or have other CVD risk factors.” The current model for QRISK2 and the models presented in this paper allow calculation of cardiovascular risk for patients with type 1 diabetes. The performance among patients with type 1 diabetes is good (see table 6 in the web appendix). We can see no reason why patients with type 1 diabetes should not have similar discussions to other patients regarding the risks and benefits of interventions. Use of the calculator in patients with type 1 diabetes is intended to allow better information to be shared with such patients on their cardiovascular risk profile. It may identify patients with a risk under 10% who may not want to take statins as well as facilitate a discussion on a range of interventions to reduce risk, including weight loss, blood pressure control, and smoking cessation. The performance of the models in patients with type 2 diabetes was lower than for patients with type 1 diabetes (for example in men with type 2 diabetes Harrell’s C=0.70, R^2^=22.9% compared with Harrell’s C=0.80, R^2^=45.6% in men with type 1 diabetes).

### Blood pressure variability

Recent studies have suggested that higher blood pressure variability is associated with increased risks of stroke^26^ and other cardiovascular events.^50^ This may be independent of mean blood pressure values,^50^ although the increased risk of cardiovascular events associated with blood pressure variability in the recent meta-analysis by Stevens et al was based on one study of 8811 patients aged more than 55 years with type 2 diabetes.^51^ In our study, both the most recent value at baseline and the standard deviation of systolic blood pressure were independently associated with increased risk of cardiovascular disease, although the addition of the standard deviation to the model did not improve discrimination or calibration. It may be difficult to implement the model with blood pressure variability in a setting where there is no historical information on blood pressure available, such as with a web calculator. While the performance and reclassification statistics suggest that its inclusion will not make a major difference at a population level, there may be some benefit from taking this factor into account for those patients with highly variable blood pressure.

### Erectile dysfunction

The true prevalence of erectile dysfunction is difficult to determine, and estimates range from 1% to 100% depending on the age of the population and how the diagnosis was made.^24^ Our study indicated that erectile dysfunction affected 2.3% of men, but this is likely to be an underestimate as it includes only men who present to their doctor with the condition and have the diagnosis or treatment recorded on their electronic record. We showed that erectile dysfunction is likely to be an independent risk factor for cardiovascular disease and was associated with a 25% increased risk of cardiovascular disease (at the mean age), which is compatible with the findings of a meta-analysis that examined the association between erectile dysfunction and cardiovascular disease risk in 13 studies.^23^ While the overall relative risk estimate from these studies was 1.44, the 95% confidence interval was broad (1.27 to 1.63) and there was substantial heterogeneity across the studies. The association was reduced to 1.34 (1.17 to 1.54) when only high quality studies were included. Our definition and others only provide a summary effect and it should be recognised that the causes of erectile dysfunction are usually a combination of the physiological and psychological and that men with vascular causes are likely to be at higher risk of cardiovascular disease than those for whom the cause is largely psychological.

### HIV/AIDS

Data from large cohorts have reported that people infected with HIV have approximately 50% greater risk of acute myocardial infarction and stroke compared with those without HIV,^52^ which may be related to antiretroviral treatment.^53^ While we found a tendency towards an increased risk of cardiovascular disease among people with HIV/AIDS this did not reach statistical significance at the 0.01 level so was not included in the final models. These results may reflect the relatively small numbers with HIV/AIDS recorded on the general practice clinical system. Also, people with HIV/AIDS tend to be younger and so have low absolute event rates and shorter periods of follow-up with an individual general practice, which may tend to underestimate the long term association. People with HIV/AIDS may receive healthcare (and prescriptions for antiretroviral treatment) from specialist clinics rather than general practices, which may explain why there are few prescriptions recorded for antiretroviral treatment on the QResearch database. Over time the recording of HIV/AIDS and prescribing of antiretroviral treatment may increase and so it is important to reassess the suitability of HIV/AIDS for inclusion in QRISK3 periodically to ensure that affected people have accurate cardiovascular risk assessments.

### Comparison with the original version of QRISK2, 2008

Our new models are well calibrated when applied to a separate validation cohort and have high levels of discrimination. We found an improvement in performance from all three models over the original version of QRISK2 from 2008,^28^ although some of this improvement is likely to be owing to the wider age range (25-84 compared with 35-74 years). Since 2008, improvements have been made to the underlying QResearch database used to derive the QRISK algorithm, which may have resulted in improvements to the performance of the algorithm over and above extending the age range from 35-74 to 25-84 years and the inclusion of additional variables. Ascertainment of cardiovascular events has improved with the linkage of the QResearch database to both Office for National Statistics mortality and Hospital Episode Statistics since 1998. The number of practices contributing to the database has more than doubled, from 531 in 2008 to over 1300. The size of the derivation cohort has increased fivefold, with 363 565 cardiovascular events arising from 50.8 million person years of observation compared with 96 709 events arising from 10.9 million person years in 2008. The recording of self assigned ethnic origin has increased; 25% in 2008 compared with 62% in the current derivation cohort. As a result of these factors, there are many more events within each ethnic group—for example, there has been a 10-fold increase in the number of cardiovascular events for non-white ethnic groups compared with 2008. This is reflected in the more accurate hazard ratios with tighter confidence intervals and improved performance statistics.

### Strengths and limitations of this study

The methods used to derive and validate these models are broadly the same as for a range of other clinical risk prediction tools derived from the QResearch database.^28^ ^54^ ^55^ ^56^ ^57^ The strengths and limitations of the approach have already been discussed in detail.^8^ ^54^ ^57^ ^58^ ^59^ ^60^ In summary, key strengths include size, duration of follow up, representativeness, and lack of selection, recall, and respondent bias. UK general practices have good levels of accuracy and completeness in recording clinical diagnoses and prescribed drugs.^61^ We think our study has good face validity since it has been conducted in the setting where most patients in the UK are assessed, treated, and followed up. Limitations of our study include the lack of formal adjudication of diagnoses, information bias, and potential for bias owing to missing data. Our database has linked hospital and mortality records for nearly all patients and is therefore likely to have picked up the majority of cardiovascular events thereby minimising ascertainment bias. We excluded patients using statins at baseline as in previous versions of QRISK and QRISK2. Over the past decade a change in guidelines will have led to a higher proportion of at risk patients being prescribed statins in the absence of established cardiovascular disease. Removing patients at high risk will tend to lower overall event rates. We excluded patients without a valid deprivation score since this group may represent a more transient population, where follow-up could be unreliable or unrepresentative. Their deprivation scores are unlikely to be missing at random so we did not think it would be appropriate to impute them. Given the number tested for inclusion, there may be some over fitting of interaction terms. We have continued to use the well recognised total cholesterol: high density lipoprotein cholesterol ratio as a predictor rather than low density lipoprotein cholesterol values alone as the ratio resulted in improved prediction during earlier versions of QRISK and QRISK2 and is measured directly, whereas low density lipoprotein cholesterol is calculated.

The present validation has been done on a separate set of practices and individuals to those that were used to develop the score, although the practices all use the same general practice clinical computer system (EMIS, used by 55% of UK general practices). An independent validation study would be a more stringent test and should be done, but when such independent studies have examined QRISK2 and other risk algorithms,^6^ ^7^ ^59^ ^60^ they have shown comparable performance compared with the validation in the QResearch database.^28^ ^54^ ^58^ We have published the source code to enable accurate implementation of QRISK3 on the QRISK website (www.qrisk.org) with earlier versions of the score from previous annual updates. The rationale for this is to ensure that those interested in reviewing or using the open source will then be able to find the current version as the score continues to be updated.

### Conclusion

We have developed updated algorithms (QRISK3) to quantify absolute risks of cardiovascular disease in people aged 25-84 years, which include established risk factors and new risk factors: expanded definition of chronic kidney disease (stage 3, 4, or 5), migraine, corticosteroid use, SLE, atypical antipsychotic use, severe mental illness, erectile dysfunction, and a measure of blood pressure variability (standard deviation of repeated measures). The updated risk algorithms provide valid measures of absolute risk in the general population of patients, as shown by the performance in a separate validation cohort.

**What is already known on this topic**

Methods to identify patients at increased risk of cardiovascular disease (CVD) are needed to identify those for whom interventions or more frequent assessment may be required
QRISK2 algorithms are widely used to estimate the 10 year risks of CVD in people aged 25-84 taking account of information recorded in primary care electronic records and that the patient can also provide

**What this study adds**

Updated algorithms (QRISK3) quantify the absolute risks of CVD in people aged 25-84, which include established and new risk factors
New factors are an expanded definition of chronic kidney disease (stage 3, 4, or 5), migraine, corticosteroid use, systemic lupus erythematosus, atypical antipsychotic use, severe mental illness, erectile dysfunction, and a measure of blood pressure variability (standard deviation of repeated measures)
The updated risk algorithms provide valid measures of absolute risk in the general population of patients as shown by the performance in a separate validation cohort

## Web Extra

Extra material supplied by the author

## Notes

A simple web calculator to implement the QRISK3 algorithms can be accessed at www.qrisk.org/Open source software is also available for download. We thank the EMIS practices that contribute to QResearch, and EMIS and the University of Nottingham for expertise in establishing, developing, and supporting the QResearch database, and the Office for National Statistics for providing the mortality data. The Hospital Episode Statistics data in this analysis are reused by permission from NHS Digital, which retains the copyright. ONS and NHS Digital bear no responsibility for the analysis or interpretation of the data.

Ethical approval: The study was reviewed in accordance with the QResearch agreement with East Midlands-Derby Research Ethics Committee (reference 03/4/021).

Data sharing: The algorithms presented in this paper will be released as open source software under the GNU lesser GPL v3. The open source software allows use without charge under the terms of the GNU lesser public license version 3. Closed source software can be licensed at a fee.

Transparency: The lead author (JHC) affirms that the manuscript is an honest, accurate, and transparent account of the study being reported; that no important aspects of the study have been omitted; and that any discrepancies from the study as planned (and, if relevant, registered) have been explained.
