# Supplementary material for: Development and validation of QRISK3 risk prediction algorithms to estimate future risk of cardiovascular disease: prospective cohort study
Source: BMJ. 2017 May 24;357:j2099. doi: 10.1136/bmj.j2099 (PMC5441081; doi:10.1136/bmj.j2099)
Supplement: Supplementary file 1 — Appendix: Supplementary materials [file hipj036510.ww.pdf]

Supplementary table 1 Read codes used to identify patients with cardiovascular disease from GP records.

Group Name	ReadTerm	ReadDesc
Coronary Heart Disease	G3	Ischaemic heart disease
Coronary Heart Disease	G3-1	Arteriosclerotic heart disease
Coronary Heart Disease	G3-2	Atherosclerotic heart disease
Coronary Heart Disease	G3-3	IHD - Ischaemic heart disease
Coronary Heart Disease	G30	Acute myocardial infarction
Coronary Heart Disease	G30-1	Attack - heart
Coronary Heart Disease	G30-2	Coronary thrombosis
Coronary Heart Disease	G30-3	Cardiac rupture following myocardial infarction (MI)
Coronary Heart Disease	G30-4	Heart attack
Coronary Heart Disease	G30-5	MI - acute myocardial infarction
Coronary Heart Disease	G30-6	Thrombosis - coronary
Coronary Heart Disease	G30-7	Silent myocardial infarction
Coronary Heart Disease	G30-98	Coronary thrombosis
Coronary Heart Disease	G30-99	Myocardial Infarction
Coronary Heart Disease	G300	Acute anterolateral infarction
Coronary Heart Disease	G301	Other specified anterior myocardial infarction
Coronary Heart Disease	G3010	Acute anteroapical infarction
Coronary Heart Disease	G3011	Acute anteroseptal infarction
Coronary Heart Disease	G301z	Anterior myocardial infarction NOS
Coronary Heart Disease	G302	Acute inferolateral infarction
Coronary Heart Disease	G303	Acute inferoposterior infarction
Coronary Heart Disease	G304	Posterior myocardial infarction NOS
Coronary Heart Disease	G305	Lateral myocardial infarction NOS
Coronary Heart Disease	G306	True posterior myocardial infarction
Coronary Heart Disease	G307	Acute subendocardial infarction
Coronary Heart Disease	G3070	Acute non-Q wave infarction
Coronary Heart Disease	G3071	Acute non-ST segment elevation myocardial infarction
Coronary Heart Disease	G308	Inferior myocardial infarction NOS
Coronary Heart Disease	G309	Acute Q-wave infarct
Coronary Heart Disease	G30A	Mural thrombosis
Coronary Heart Disease	G30B	Acute posterolateral myocardial infarction
Coronary Heart Disease	G30X	Acute transmural myocardial infarction of unspecif site
Coronary Heart Disease	G30X0	Acute ST segment elevation myocardial infarction
Coronary Heart Disease	G30y	Other acute myocardial infarction
Coronary Heart Disease	G30y0	Acute atrial infarction
Coronary Heart Disease	G30y1	Acute papillary muscle infarction
Coronary Heart Disease	G30y2	Acute septal infarction
Coronary Heart Disease	G30yz	Other acute myocardial infarction NOS
Coronary Heart Disease	G30z	Acute myocardial infarction NOS
Coronary Heart Disease	G31	Other acute and subacute ischaemic heart disease
Coronary Heart Disease	G31-99	Acute/subacute IHD NOS
Coronary Heart Disease	G310	Postmyocardial infarction syndrome

Coronary Heart Disease	G310-1	Dressler's syndrome
Coronary Heart Disease	G311	Preinfarction syndrome
Coronary Heart Disease	G311-1	Crescendo angina
Coronary Heart Disease	G311-2	Impending infarction
Coronary Heart Disease	G311-3	Unstable angina
Coronary Heart Disease	G311-4	Angina at rest
Coronary Heart Disease	G3110	Myocardial infarction aborted
Coronary Heart Disease	G3110-1	MI - myocardial infarction aborted
Coronary Heart Disease	G3111	Unstable angina
Coronary Heart Disease	G3112	Angina at rest
Coronary Heart Disease	G3113	Refractory angina
Coronary Heart Disease	G3114	Worsening angina
Coronary Heart Disease	G3115	Acute coronary syndrome
Coronary Heart Disease	G311z	Preinfarction syndrome NOS
Coronary Heart Disease	G312	Coronary thrombosis not resulting in myocardial infarction
Coronary Heart Disease	G31y	Other acute and subacute ischaemic heart disease
Coronary Heart Disease	G31y0	Acute coronary insufficiency
Coronary Heart Disease	G31y0-99	Acute coronary syndrome
Coronary Heart Disease	G31y1	Microinfarction of heart
Coronary Heart Disease	G31y2	Subendocardial ischaemia
Coronary Heart Disease	G31y3	Transient myocardial ischaemia
Coronary Heart Disease	G31yz	Other acute and subacute ischaemic heart disease NOS
Coronary Heart Disease	G32	Old myocardial infarction
Coronary Heart Disease	G32-1	Healed myocardial infarction
Coronary Heart Disease	G32-2	Personal history of myocardial infarction
Coronary Heart Disease	G33	Angina pectoris
Coronary Heart Disease	G330	Angina decubitus
Coronary Heart Disease	G3300	Nocturnal angina
Coronary Heart Disease	G330z	Angina decubitus NOS
Coronary Heart Disease	G331	Prinzmetal's angina
Coronary Heart Disease	G331-1	Variant angina pectoris
Coronary Heart Disease	G332	Coronary artery spasm
Coronary Heart Disease	G33z	Angina pectoris NOS
Coronary Heart Disease	G33z0	Status anginosus
Coronary Heart Disease	G33z1	Stenocardia
Coronary Heart Disease	G33z2	Syncope anginosa
Coronary Heart Disease	G33z3	Angina on effort
Coronary Heart Disease	G33z4	Ischaemic chest pain
Coronary Heart Disease	G33z5	Post infarct angina
Coronary Heart Disease	G33z6	New onset angina
Coronary Heart Disease	G33z7	Stable angina
Coronary Heart Disease	G33zz	Angina pectoris NOS
Coronary Heart Disease	G34	Other chronic ischaemic heart disease
Coronary Heart Disease	G34-99	Chr. ischaemic heart dis. NOS

Coronary Heart Disease	G340	Coronary atherosclerosis
Coronary Heart Disease	G340-1	Triple vessel disease of the heart
Coronary Heart Disease	G340-2	Coronary artery disease
Coronary Heart Disease	G3400	Single coronary vessel disease
Coronary Heart Disease	G3401	Double coronary vessel disease
Coronary Heart Disease	G342	Atherosclerotic cardiovascular disease
Coronary Heart Disease	G343	Ischaemic cardiomyopathy
Coronary Heart Disease	G344	Silent myocardial ischaemia
Coronary Heart Disease	G34y	Other specified chronic ischaemic heart disease
Coronary Heart Disease	G34y0	Chronic coronary insufficiency
Coronary Heart Disease	G34y1	Chronic myocardial ischaemia
Coronary Heart Disease	G34yz	Other specified chronic ischaemic heart disease NOS
Coronary Heart Disease	G34z	Other chronic ischaemic heart disease NOS
Coronary Heart Disease	G34z0	Asymptomatic coronary heart disease
Coronary Heart Disease	G35	Subsequent myocardial infarction
Coronary Heart Disease	G350	Subsequent myocardial infarction of anterior wall
Coronary Heart Disease	G351	Subsequent myocardial infarction of inferior wall
Coronary Heart Disease	G353	Subsequent myocardial infarction of other sites
Coronary Heart Disease	G35X	Subsequent myocardial infarction of unspecified site
Coronary Heart Disease	G36	Certain current complication follow acute myocardial infarct
Coronary Heart Disease	G360	Haemopericardium/current comp folow acut myocard infarct
Coronary Heart Disease	G361	Atrial septal defect/curr comp folow acut myocardal infarct
Coronary Heart Disease	G362	Ventric septal defect/curr comp fol acut myocardal infarctn
Coronary Heart Disease	G363	Ruptur cardiac wall w'out haemopericard/cur comp fol ac MI
Coronary Heart Disease	G364	Ruptur chordae tendinae/curr comp fol acute myocard infarct
Coronary Heart Disease	G365	Rupture papillary muscle/curr comp fol acute myocard infarct
Coronary Heart Disease	G366	Thrombosis atrium,auric append&vent/curr comp foll acute MI
Coronary Heart Disease	G38	Postoperative myocardial infarction
Coronary Heart Disease	G380	Postoperative transmural myocardial infarction anterior wall
Coronary Heart Disease	G381	Postoperative transmural myocardial infarction inferior wall
Coronary Heart Disease	G382	Postoperative transmural myocardial infarction other sites
Coronary Heart Disease	G383	Postoperative transmural myocardial infarction unspec site
Coronary Heart Disease	G384	Postoperative subendocardial myocardial infarction
Coronary Heart Disease	G38z	Postoperative myocardial infarction, unspecified
Coronary Heart Disease	G3y	Other specified ischaemic heart disease
Coronary Heart Disease	G3z	Ischaemic heart disease NOS
Coronary Heart Disease	G501	Post infarction pericarditis
Coronary Heart Disease	Gyu34	[X]Acute transmural myocardial infarction of unspecif site
Stroke or TIA	F4236	Amaurosis fugax
Stroke or TIA	Fyu55	[X]Other transnt cerebral ischaemic attacks+related syndroms
Stroke or TIA	G63y0	Cerebral infarct due to thrombosis of precerebral arteries
Stroke or TIA	G63y1	Cerebral infarction due to embolism of precerebral arteries
Stroke or TIA	G64	Cerebral arterial occlusion
Stroke or TIA	G64-1	CVA - cerebral artery occlusion

Stroke or TIA	G64-2	Infarction - cerebral
Stroke or TIA	G64-3	Stroke due to cerebral arterial occlusion
Stroke or TIA	G640	Cerebral thrombosis
Stroke or TIA	G6400	Cerebral infarction due to thrombosis of cerebral arteries
Stroke or TIA	G641	Cerebral embolism
Stroke or TIA	G641-1	Cerebral embolus
Stroke or TIA	G6410	Cerebral infarction due to embolism of cerebral arteries
Stroke or TIA	G64z	Cerebral infarction NOS
Stroke or TIA	G64z-1	Brainstem infarction NOS
Stroke or TIA	G64z-2	Cerebellar infarction
Stroke or TIA	G64z-99	Cerebral A. occlusion NOS
Stroke or TIA	G64z0	Brainstem infarction
Stroke or TIA	G64z1	Wallenberg syndrome
Stroke or TIA	G64z1-1	Lateral medullary syndrome
Stroke or TIA	G64z2	Left sided cerebral infarction
Stroke or TIA	G64z3	Right sided cerebral infarction
Stroke or TIA	G64z4	Infarction of basal ganglia
Stroke or TIA	G65	Transient cerebral ischaemia
Stroke or TIA	G65-1	Drop attack
Stroke or TIA	G65-2	Transient ischaemic attack
Stroke or TIA	G65-3	Vertebro-basilar insufficiency
Stroke or TIA	G65-99	Transient Ischaemic Attacks
Stroke or TIA	G650	Basilar artery syndrome
Stroke or TIA	G650-1	Insufficiency - basilar artery
Stroke or TIA	G652	Subclavian steal syndrome
Stroke or TIA	G653	Carotid artery syndrome hemispheric
Stroke or TIA	G654	Multiple and bilateral precerebral artery syndromes
Stroke or TIA	G656	Vertebrobasilar insufficiency
Stroke or TIA	G65y	Other transient cerebral ischaemia
Stroke or TIA	G65z	Transient cerebral ischaemia NOS
Stroke or TIA	G65z-99	Transient Ischaemic Attacks
Stroke or TIA	G65z0	Impending cerebral ischaemia
Stroke or TIA	G65z1	Intermittent cerebral ischaemia
Stroke or TIA	G65zz	Transient cerebral ischaemia NOS
Stroke or TIA	G66	Stroke and cerebrovascular accident unspecified
Stroke or TIA	G66-1	CVA unspecified
Stroke or TIA	G66-2	Stroke unspecified
Stroke or TIA	G66-3	CVA - Cerebrovascular accident unspecified
Stroke or TIA	G66-98	Stroke/CVA - undefined
Stroke or TIA	G66-99	Stroke
Stroke or TIA	G667	Left sided CVA
Stroke or TIA	G668	Right sided CVA
Stroke or TIA	G6760	Cereb infarct due cerebral venous thrombosis, nonpyogenic
Stroke or TIA	G6W	Cereb infarct due unsp occlus/stenos precerebr arteries

Stroke or TIA	G6X	Cerebrl infarctn due/unspcf occlusn or sten/cerebrl artrs
Stroke or TIA	Gyu63	[X]Cerebrl infarctn due/unspcf occlusn or sten/cerebrl artrs
Stroke or TIA	Gyu64	[X]Other cerebral infarction
Stroke or TIA	Gyu65	[X]Occlusion and stenosis of other precerebral arteries
Stroke or TIA	Gyu66	[X]Occlusion and stenosis of other cerebral arteries
Stroke or TIA	ZV12D	[V]Personal history of transient ischaemic attack

Supplementary table 2: number of cardiovascular events and person years by ethnic group in the derivation cohort. Incidence rates are directly standardised by 5-year age bands.

	women			men			
	**CVD cases**	**pyrs**	**age standardised rate per 10,000 pyrs**	**CVD cases**	**age standardised rate per 10,000 pyrs**	**total cases**	**total pyrs**
White/not recorded	153299	23780308	61.65 (58.6 to 64.71)	192246	76.98 (73.48 to 80.49)	345545	46576770
Indian	1854	413128	61.39 (29.09 to 93.7)	2904	85.93 (51.77 to 120.1)	4758	819177
Pakistani	1150	223064	112.74 (54.96 to 170.52)	1842	72.65 (20.06 to 125.24)	2992	463296
Bangladeshi	664	170068	126.03 (6.73 to 245.33)	1483	78.96 (14.73 to 143.19)	2147	374216
Other Asian	602	225039	28.63 (0 to 60.15)	908	37.04 (8.01 to 66.06)	1510	418524
Caribbean	1290	267185	82.06 (35.86 to 128.26)	1356	70.99 (25.29 to 116.68)	2646	475176
Black african	509	319042	17.02 (0 to 34.75)	729	33.58 (0 to 69.69)	1238	615099
Chinese	175	116629	35.6 (29.4 to 41.8)	242	46.9 (39.4 to 54.4)	417	210267
Other	1006	428773	71.95 (0 to 145.39)	1306	55.08 (20.06 to 90.1)	2312	812342

Supplementary table 3: type and data source for CVD incident event. Counts represent the data source which first identified the type of event. The 2 last columns shows the number and % of all events which were only identified on GP data (ie not subsequently identified on HES or ONS mortality data)

event type	**GP data**	**col %**	**HES data**	**col%**	**ONS data**	**col%**	**total**	**col %**	**GP data only**	**% events on GP data only (row %)**
myocardial infarct	17719	10.7	48675	31.3	11933	27.9	78327	21.5	6,361	8.1
angina	74645	45.2	53201	34.2	24295	56.8	152141	41.8	34463	22.7
TIA	36123	21.9	13368	8.6	13	0.0	49504	13.6	27,227	55.0
ischaemic stroke	36622	22.2	40423	26.0	6548	15.3	83593	23.0	24,885	29.8
Total	165109	100.0	155667	100.0	42789	100.0	363565	100.0	92,936	25.6

Supplementary table 4 complete case analysis men for models B and C. see table 4 for fractional polynomial terms and interactions. Model B Complete case 1 includes the closest cholesterol/HDL to baseline. Model B complete case 2 is restricted to cholesterol/HDL measured prior to entry; model C complete case restricted to those with 2+ systolic blood pressure readings prior to cohort entry

	**model B complete case 1 adjusted HR (95% CI)**	**model B complete case 2 adjusted HR (95% CI)**	**Model C complete case adjusted HR (95% CI)**
white or not recorded	1.00	1.00	1.00
Indian	1.54 (1.44 to 1.65)	1.26 (1.12 to 1.42)	1.69 (1.56 to 1.84)
Pakistani	2.18 (2 to 2.39)	1.81 (1.58 to 2.08)	2.13 (1.9 to 2.38)
Bangladeshi	1.78 (1.6 to 1.98)	1.3 (1.1 to 1.55)	1.65 (1.46 to 1.88)
Other Asian	1.5 (1.35 to 1.67)	1.23 (1.05 to 1.46)	1.53 (1.35 to 1.74)
Caribbean	.873 (.805 to .946)	.99 (.868 to 1.13)	.861 (.784 to .945)
Black African	.953 (.851 to 1.07)	.684 (.578 to .81)	.962 (.844 to 1.1)
Chinese	.903 (.731 to 1.12)	.477 (.292 to .78)	.707 (.526 to .95)
Other ethnicity	1.03 (.943 to 1.12)	.886 (.767 to 1.02)	1.05 (.942 to 1.16)
non-smoker	1.00	1.00	1.00
ex-smoker	1.07 (1.02 to 1.12)	.914 (.805 to 1.04)	1.07 (1.01 to 1.13)
light smoker	1.74 (1.66 to 1.82)	1.75 (1.56 to 1.96)	1.69 (1.59 to 1.79)
moderate smoker	1.92 (1.82 to 2.03)	2.03 (1.77 to 2.33)	1.88 (1.75 to 2.02)
heavy smoker	2.32 (2.19 to 2.46)	2.48 (2.13 to 2.89)	2.27 (2.1 to 2.46)
**medical history**			
atrial fibrillation	4.41 (3.44 to 5.64)	2.77 (1.34 to 5.7)	4.56 (3.42 to 6.08)
atypical antipsychotics	1.44 (1.28 to 1.62)	1.2 (1.02 to 1.41)	1.5 (1.31 to 1.72)
corticosteroids	1.55 (1.45 to 1.65)	1.64 (1.41 to 1.9)	1.5 (1.38 to 1.63)
migraine	1.39 (1.32 to 1.46)	1.35 (1.21 to 1.5)	1.39 (1.31 to 1.48)
rheumatoid arthritis	1.34 (1.28 to 1.4)	1.25 (1.15 to 1.36)	1.34 (1.27 to 1.41)
Chronic kidney disease (stages3 4 or 5)	1.64 (1.34 to 2.01)	1.76 (1.33 to 2.34)	1.74 (1.4 to 2.17)
severe mental illness	1.23 (1.19 to 1.27)	1.21 (1.15 to 1.28)	1.23 (1.18 to 1.27)

SLE	2.02 (1.53 to 2.66)	1.83 (1.07 to 3.14)	2.1 (1.51 to 2.93)
treated hypertension	1.73 (1.64 to 1.82)	1.66 (1.52 to 1.82)	1.65 (1.55 to 1.75)
type 1 diabetes	5.08 (4.45 to 5.79)	4.46 (3.56 to 5.59)	4.83 (4.17 to 5.59)
type 2 diabetes	2.82 (2.6 to 3.06)	2.32 (2.03 to 2.65)	2.68 (2.44 to 2.93)
cholesterol/HDL ratio	1.16 (1.15 to 1.16)	1.14 (1.12 to 1.15)	1.15 (1.14 to 1.16)
FH coronary heart disease	1.48 (1.43 to 1.53)	1.6 (1.48 to 1.74)	1.48 (1.41 to 1.54)
systolic blood pressure	1.11 (1.1 to 1.12)	1.11 (1.08 to 1.13)	1.09 (1.08 to 1.1)
Standard deviation of blood SBP	n/a	n/a	1.09 (1.07 to 1.1)
Townsend Score (5-unit increase)	1.4 (1.37 to 1.44)	1.34 (1.26 to 1.42)	1.4 (1.35 to 1.45)

Supplementary table 5 complete case analysis men for models B and C. see table 4 for fractional polynomial terms and interactions. Model B Complete case 1 includes the closest cholesterol/HDL to baseline. Model B complete case 2 is restricted to cholesterol/HDL measured prior to entry; model C complete case restricted to those with 2+ systolic blood pressure readings prior to cohort entry

	**Model B complete case 1 Adjusted HR (95% CI)**	**Model B complete case 2 Adjusted HR (95% CI)**	**Model C complete case Adjusted HR (95% CI)**
White or not recorded	1.00	1.00	1.00
Indian	1.49 (1.4 to 1.57)	1.25 (1.14 to 1.37)	1.37 (1.27 to 1.48)
Pakistani	1.9 (1.77 to 2.04)	1.63 (1.46 to 1.82)	1.79 (1.62 to 1.98)
Bangladeshi	1.93 (1.79 to 2.08)	1.3 (1.13 to 1.48)	1.7 (1.53 to 1.89)
Other Asian	1.25 (1.14 to 1.37)	1.11 (.972 to 1.28)	1.26 (1.12 to 1.42)
Caribbean	.747 (.689 to .81)	.661 (.575 to .759)	.734 (.664 to .811)
Black African	.836 (.758 to .922)	.598 (.516 to .694)	.824 (.73 to .931)
Chinese	.894 (.743 to 1.08)	.625 (.449 to .872)	.931 (.728 to 1.19)
Other ethnicity	.916 (.846 to .992)	.79 (.695 to .896)	.936 (.845 to 1.04)
Non-smoker	1.00	1.00	1.00
ex-smoker	1.13 (1.08 to 1.17)	1.2 (1.1 to 1.31)	1.15 (1.08 to 1.22)
light smoker	1.66 (1.6 to 1.72)	1.9 (1.75 to 2.06)	1.67 (1.58 to 1.77)
moderate smoker	1.82 (1.74 to 1.9)	1.92 (1.73 to 2.14)	1.77 (1.64 to 1.9)
heavy smoker	2.09 (2.01 to 2.18)	2.7 (2.44 to 2.98)	2.13 (1.99 to 2.28)
**Medical history**			
atrial fibrillation	2.29 (1.89 to 2.77)	1.94 (1.34 to 2.79)	2.37 (1.87 to 3.01)
atypical antipsychotics	1.02 (.902 to 1.15)	.913 (.772 to 1.08)	.96 (.827 to 1.11)
corticosteroids	1.39 (1.29 to 1.5)	1.35 (1.1 to 1.66)	1.33 (1.19 to 1.5)
erectile	1.64 (1.52 to 1.77)	1.35 (1.21 to 1.51)	1.62 (1.47 to 1.78)
migraine	1.26 (1.18 to 1.34)	1.25 (1.1 to 1.41)	1.2 (1.11 to 1.31)
rheumatoid arthritis	1.31 (1.23 to 1.38)	1.21 (1.09 to 1.34)	1.27 (1.18 to 1.37)

CKD (stages3, 4 or 5)	1.77 (1.47 to 2.14)	1.74 (1.27 to 2.39)	1.76 (1.41 to 2.19)
severe mental illness	1.25 (1.21 to 1.29)	1.13 (1.06 to 1.2)	1.25 (1.20 to 1.31)
SLE	1.06 (.585 to 1.91)	3.36 (1.6 to 7.05)	.911 (.409 to 2.03)
treated hypertension	1.66 (1.59 to 1.75)	1.72 (1.58 to 1.86)	1.41 (1.33 to 1.49)
type 1 diabetes	2.99 (2.68 to 3.34)	2.29 (1.86 to 2.81)	2.51 (2.22 to 2.85)
type 2 diabetes	2.13 (1.99 to 2.29)	1.78 (1.60 to 1.99)	1.89 (1.74 to 2.05)
TSC/HDL ratio	1.19 (1.18 to 1.19)	1.17 (1.15 to 1.18)	1.17 (1.16 to 1.18)
FH CHD	1.46 (1.42 to 1.50)	1.55 (1.45 to 1.65)	1.40 (1.34 to 1.46)
systolic blood pressure	1.10 (1.09 to 1.11)	1.11 (1.09 to 1.14)	1.10 (1.08 to 1.11)
SD of blood pressure	n/a	n/a	1.09 (1.08 to 1.11)
Townsend Score (5-unit increase)	1.22 (1.2 to 1.25)	1.14 (1.09 to 1.2)	1.22 (1.18 to 1.26)

Supplementary table 6 performance of model B in the validation cohort in men and women by sub-groups

Sub-group	statistic	Women mean (95% CI)	men mean (95% CI)
< 40 years	D statistic	1.66 (1.55 to 1.76)	1.75 (1.69 to 1.82)
	Harrell's C	.747 (.728 to .766)	.781 (.771 to .792)
	R^2	39.6 (36.5 to 42.7)	42.3 (40.4 to 44.1)
40-59 years	D statistic	1.48 (1.44 to 1.51)	1.33 (1.31 to 1.36)
	Harrell's C	.752 (.747 to .757)	.732 (.728 to .736)
	R^2	34.3 (33.2 to 35.4)	29.8 (29 to 30.5)
60+ years	D statistic	1.11 (1.09 to 1.13)	.903 (.883 to .922)
	Harrell's C	.692 (.689 to .695)	.659 (.656 to .663)
	R^2	22.7 (22 to 23.4)	16.3 (15.7 to 16.9)
White or not recorded	D statistic	2.46 (2.44 to 2.47)	2.23 (2.22 to 2.25)
	Harrell's C	.877 (.876 to .879)	.855 (.854 to .857)
	R^2	59 (58.6 to 59.4)	54.3 (54 to 54.7)
Indian	D statistic	2.49 (2.27 to 2.71)	2.22 (2.06 to 2.38)
	Harrell's C	.878 (.861 to .894)	.86 (.843 to .877)
	R^2	59.7 (55.5 to 63.9)	54.1 (50.6 to 57.7)
Pakistani	D statistic	2.32 (2.03 to 2.6)	2.21 (2.02 to 2.41)
	Harrell's C	.871 (.848 to .894)	.87 (.851 to .889)
	R^2	56.1 (50.1 to 62.1)	53.9 (49.6 to 58.2)
Bangladeshi	D statistic	2.43 (2.1 to 2.76)	2.38 (2.17 to 2.6)
	Harrell's C	.888 (.862 to .914)	.873 (.85 to .895)
	R2	58.4 (51.8 to 65.1)	57.5 (53.1 to 62)
Other Asian	D statistic	2.47 (2.17 to 2.77)	2.32 (2.11 to 2.53)
	Harrell's C	.876 (.845 to .908)	.875 (.858 to .893)
	R2	59.3 (53.5 to 65.1)	56.2 (51.7 to 60.7)
Caribbean	D statistic	2.11 (1.92 to 2.31)	2.13 (1.93 to 2.33)
	Harrell's C	.846 (.826 to .865)	.844 (.825 to .864)
	R2	51.6 (46.9 to 56.4)	51.9 (47.2 to 56.6)
Black african	D statistic	2.46 (2.17 to 2.75)	2.15 (1.92 to 2.38)
	Harrell's C	.857 (.822 to .892)	.86 (.839 to .881)
	R2	59.1 (53.3 to 64.8)	52.5 (47.1 to 57.8)
Chinese	D statistic	2.77 (2.18 to 3.35)	2.43 (1.95 to 2.91)
	Harrell's C	.91 (.876 to .944)	.888 (.852 to .925)
	R2	64.7 (55 to 74.3)	58.5 (48.8 to 68.1)
Other ethnicity	D statistic	2.59 (2.35 to 2.83)	2.37 (2.18 to 2.57)
	Harrell's C	.895 (.874 to .915)	.88 (.863 to .898)
	R2	61.5 (57.2 to 65.9)	57.4 (53.3 to 61.4)
atrial fibrillation	D statistic	.772 (.667 to .878)	1.02 (.93 to 1.12)
	Harrell's C	.635 (.618 to .652)	.675 (.66 to .69)
	R2	12.5 (9.49 to 15.4)	20 (17.1 to 23)

©Julia Hipisley-Cox, 2015.

atypical antipsychotics	D statistic	1.85 (1.66 to 2.05)	1.9 (1.7 to 2.1)
	Harrell's C	.829 (.808 to .849)	.831 (.807 to .854)
	R2	45.1 (39.8 to 50.4)	46.3 (41 to 51.6)
corticosteroids	D statistic	1.65 (1.59 to 1.72)	1.53 (1.46 to 1.6)
	Harrell's C	.788 (.781 to .795)	.769 (.761 to .778)
	R2	39.5 (37.7 to 41.3)	35.8 (33.7 to 38)
erectile dysfunction or treatment	D statistic	n/a	1.45 (1.38 to 1.52)
	Harrell's C	n/a	.753 (.743 to .763)
	R2	n/a	33.4 (31.2 to 35.7)
migraine	D statistic	2.18 (2.11 to 2.25)	2 (1.9 to 2.09)
	Harrell's C	.845 (.837 to .853)	.831 (.821 to .842)
	R2	53.1 (51.4 to 54.8)	48.8 (46.5 to 51.1)
rheumatoid arthritis	D statistic	1.59 (1.5 to 1.69)	1.48 (1.36 to 1.6)
	Harrell's C	.773 (.761 to .784)	.753 (.738 to .769)
	R2	37.8 (35.1 to 40.4)	34.3 (30.6 to 38)
chronic kidney disease (stages3,4 or 5)	D statistic	1.32 (1.17 to 1.47)	1.28 (1.13 to 1.44)
	Harrell's C	.742 (.72 to .764)	.737 (.715 to .76)
	R2	29.4 (24.6 to 34.1)	28.2 (23.3 to 33.1)
severe mental illness	D statistic	2.16 (2.1 to 2.22)	1.94 (1.87 to 2.02)
	Harrell's C	.844 (.837 to .851)	.817 (.809 to .825)
	R2	52.6 (51.2 to 54.1)	47.4 (45.6 to 49.3)
SLE	D statistic	1.87 (1.46 to 2.28)	2.13 (.865 to 3.39)
	Harrell's C	.788 (.74 to .835)	.824 (.733 to .915)
	R2	45.4 (34.6 to 56.3)	51.8 (22.2 to 81.4)
treated hypertension	D statistic	1.23 (1.19 to 1.26)	1.08 (1.04 to 1.11)
	Harrell's C	.71 (.706 to .715)	.688 (.682 to .693)
	R2	26.4 (25.4 to 27.5)	21.7 (20.5 to 22.9)
type 1 diabetes	D statistic	1.94 (1.66 to 2.22)	1.87 (1.64 to 2.11)
	Harrell's C	.823 (.789 to .857)	.804 (.776 to .832)
	R2	47.3 (40.2 to 54.4)	45.6 (39.4 to 51.8)
type 2 diabetes	D statistic	1.19 (1.12 to 1.25)	1.12 (1.06 to 1.17)
	Harrell's C	.701 (.691 to .711)	.696 (.687 to .704)
	R2	25.2 (23 to 27.3)	22.9 (21.2 to 24.7)
FH coronary heart disease 1st degree relative under 60	D statistic	2.11 (2.07 to 2.16)	1.82 (1.78 to 1.86)
	Harrell's C	.84 (.835 to .844)	.805 (.8 to .81)
	R2	51.6 (50.5 to 52.6)	44.3 (43.2 to 45.4)

©Julia Hipisley-Cox, 2015.

©Julia Hipisley-Cox, 2015.

©Julia Hipisley-Cox, 2015.
