# Supplementary material for: Development and validation of QRISK3 risk prediction algorithms to estimate future risk of cardiovascular disease: prospective cohort study
Source: BMJ. 2017 May 24;357:j2099. doi: 10.1136/bmj.j2099 (PMC5441081; doi:10.1136/bmj.j2099)
Supplement: Supplementary file 2 — Appendix: Supplementary figures [file hipj036510.wf1.pdf]

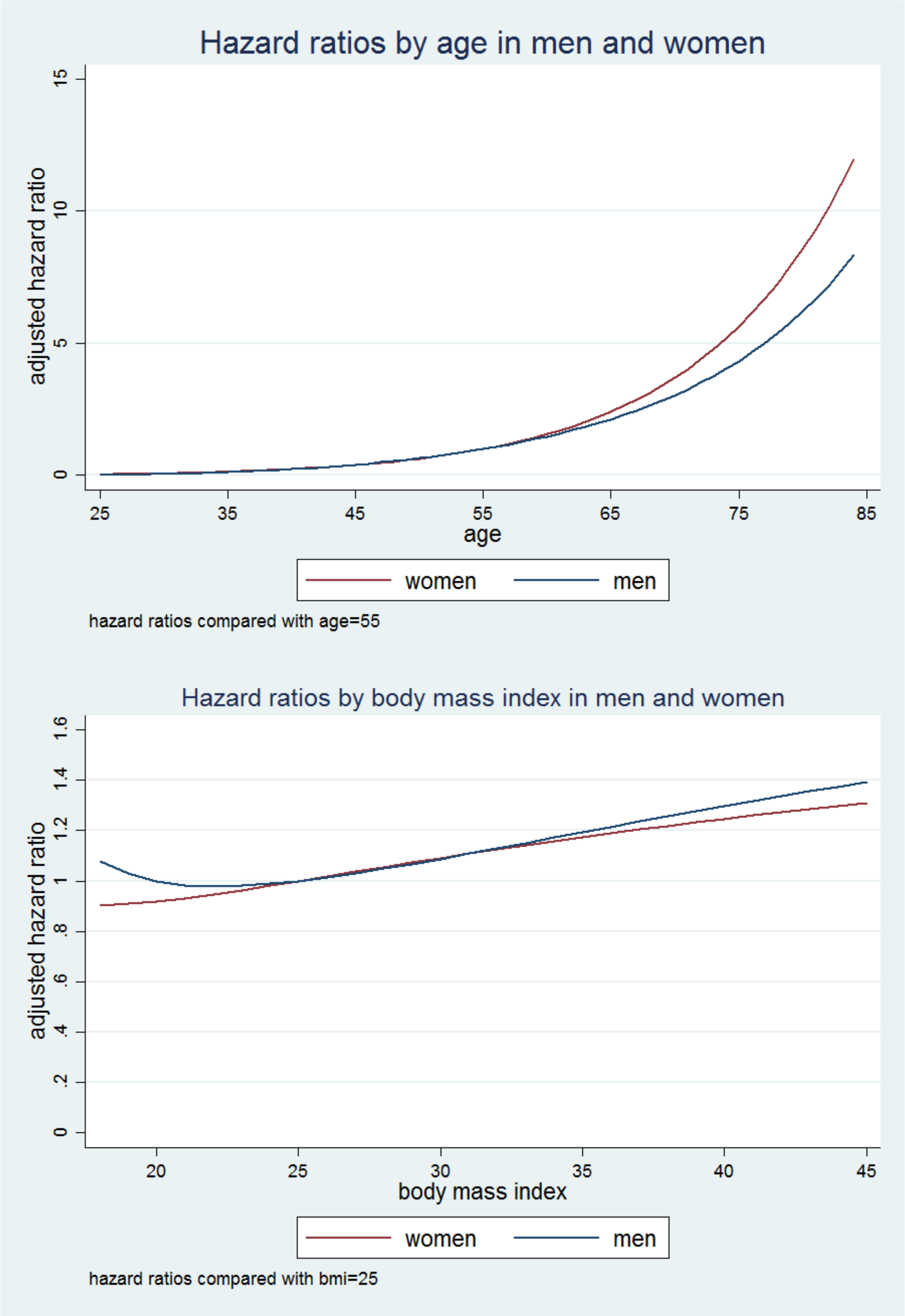

Hazard ratios by age in men and women
adjusted hazard ratio
0
5
10
15
25
35
45
55
65
75
85
age
women
men
hazard ratios compared with age=55
Hazard ratios by body mass index in men and women
adjusted hazard ratio
0
.2
.4
.6
.8
1
1.2
1.4
1.6
20
25
30
35
40
45
body mass index
women
men
hazard ratios compared with bmi=25

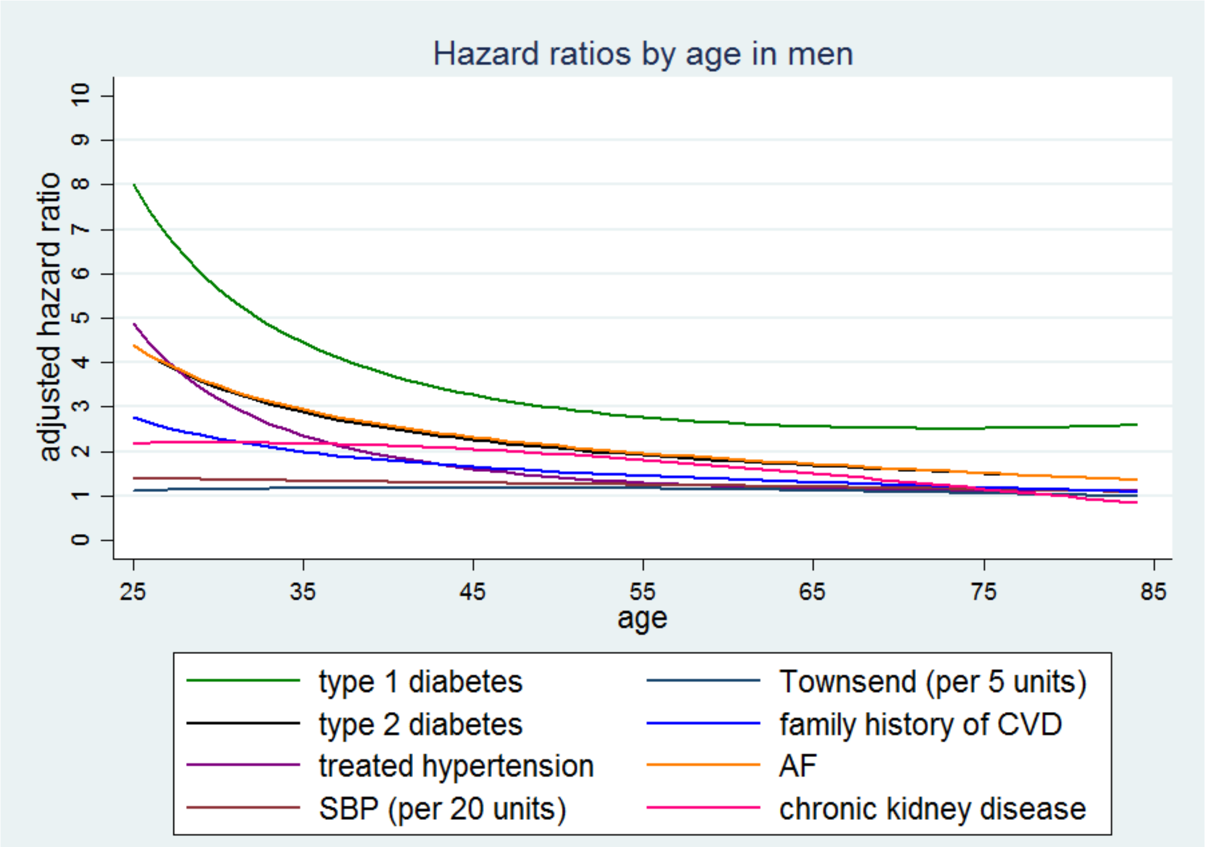
Hazard ratios by age in men
adjusted hazard ratio
0
1
2
3
4
5
6
7
8
9
10
25
35
45
55
65
75
85
age
type 1 diabetes
type 2 diabetes
treated hypertension
SBP (per 20 units)
Townsend (per 5 units)
family history of CVD
AF
chronic kidney disease

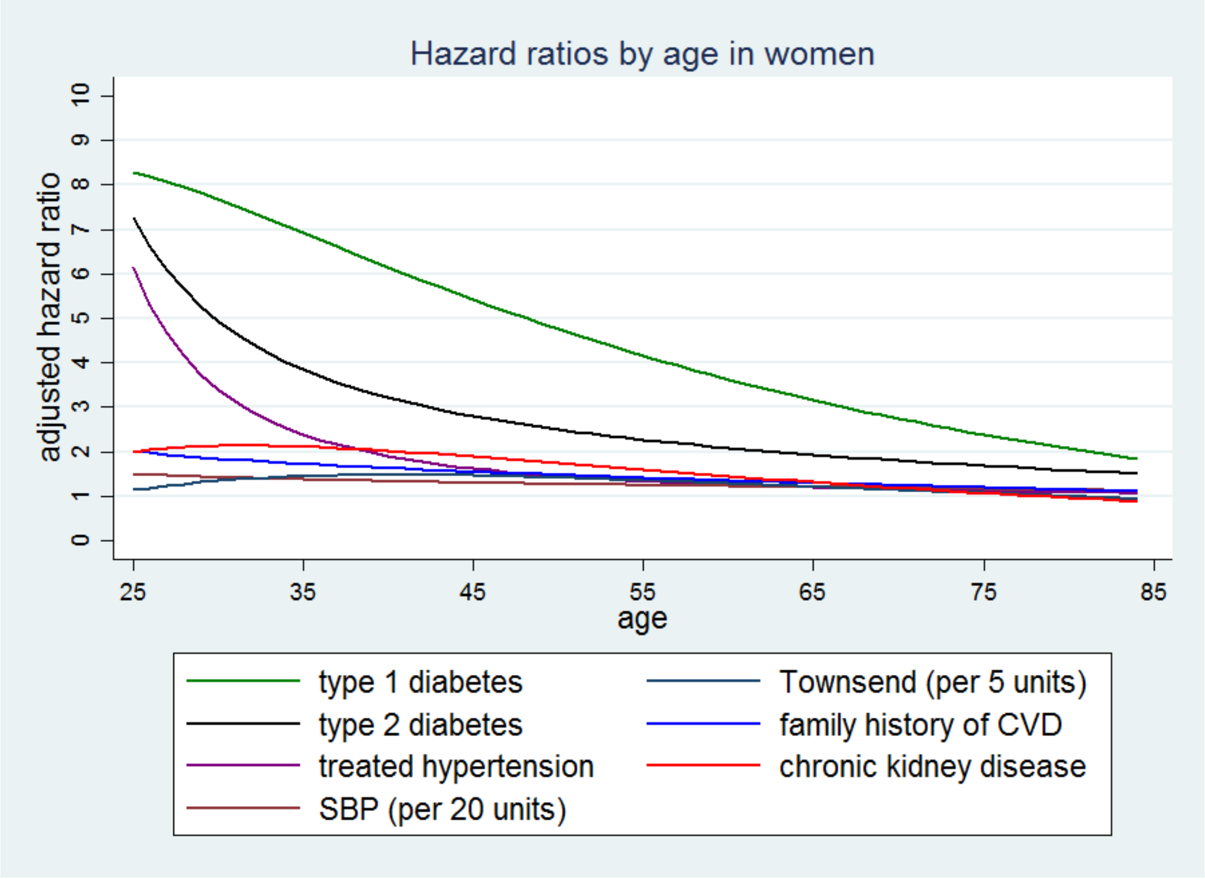
Hazard ratios by age in women
adjusted hazard ratio
0
1
2
3
4
5
6
7
8
9
10
25
35
45
55
65
75
85
age
type 1 diabetes
type 2 diabetes
treated hypertension
SBP (per 20 units)
Townsend (per 5 units)
family history of CVD
chronic kidney disease

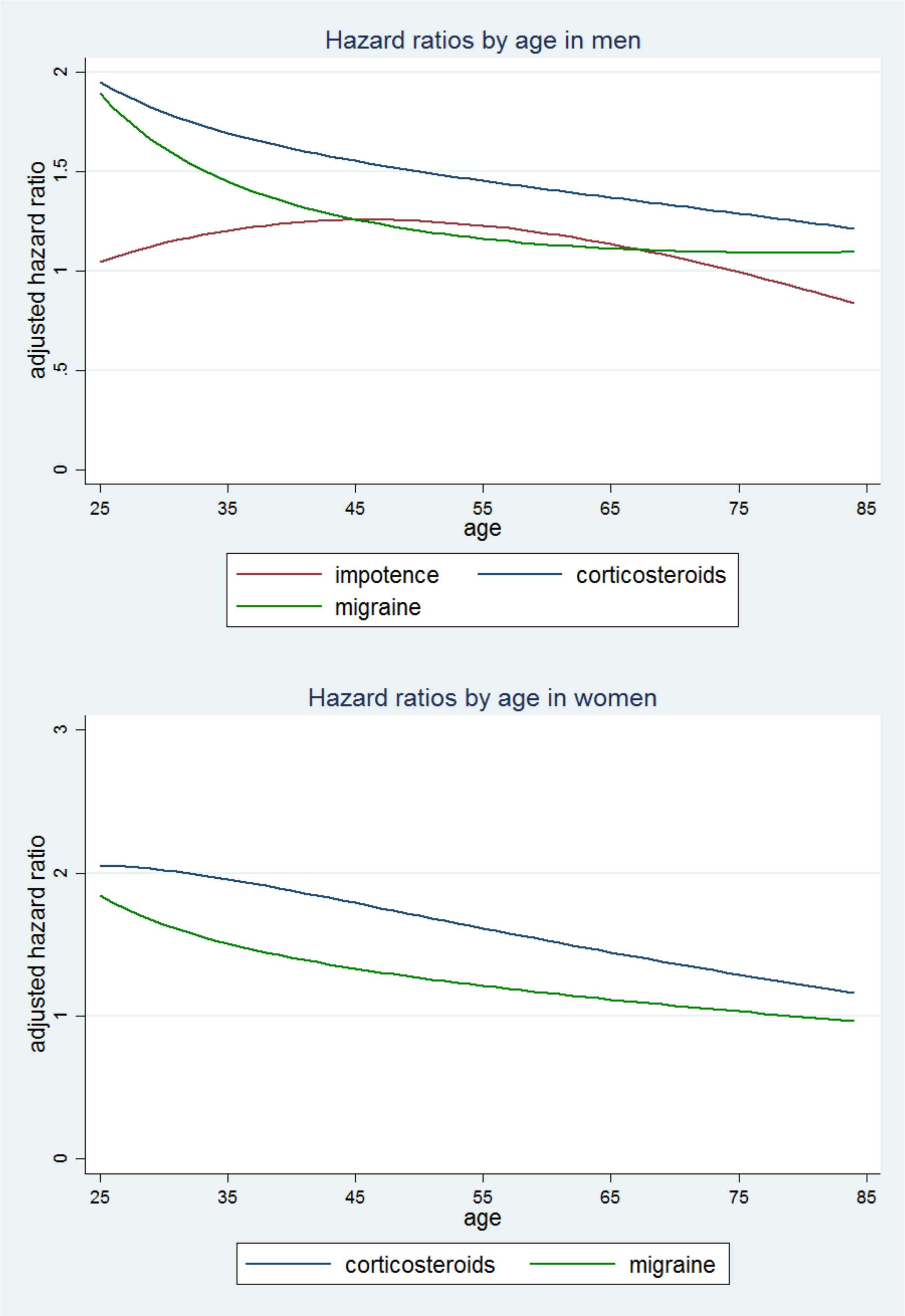
Hazard ratios by age in men
adjusted hazard ratio
0
.5
1
1.5
2
25
35
45
55
65
75
85
age
impotence
corticosteroids
migraine
Hazard ratios by age in women
adjusted hazard ratio
0
1
2
3
25
35
45
55
65
75
85
age
corticosteroids
migraine

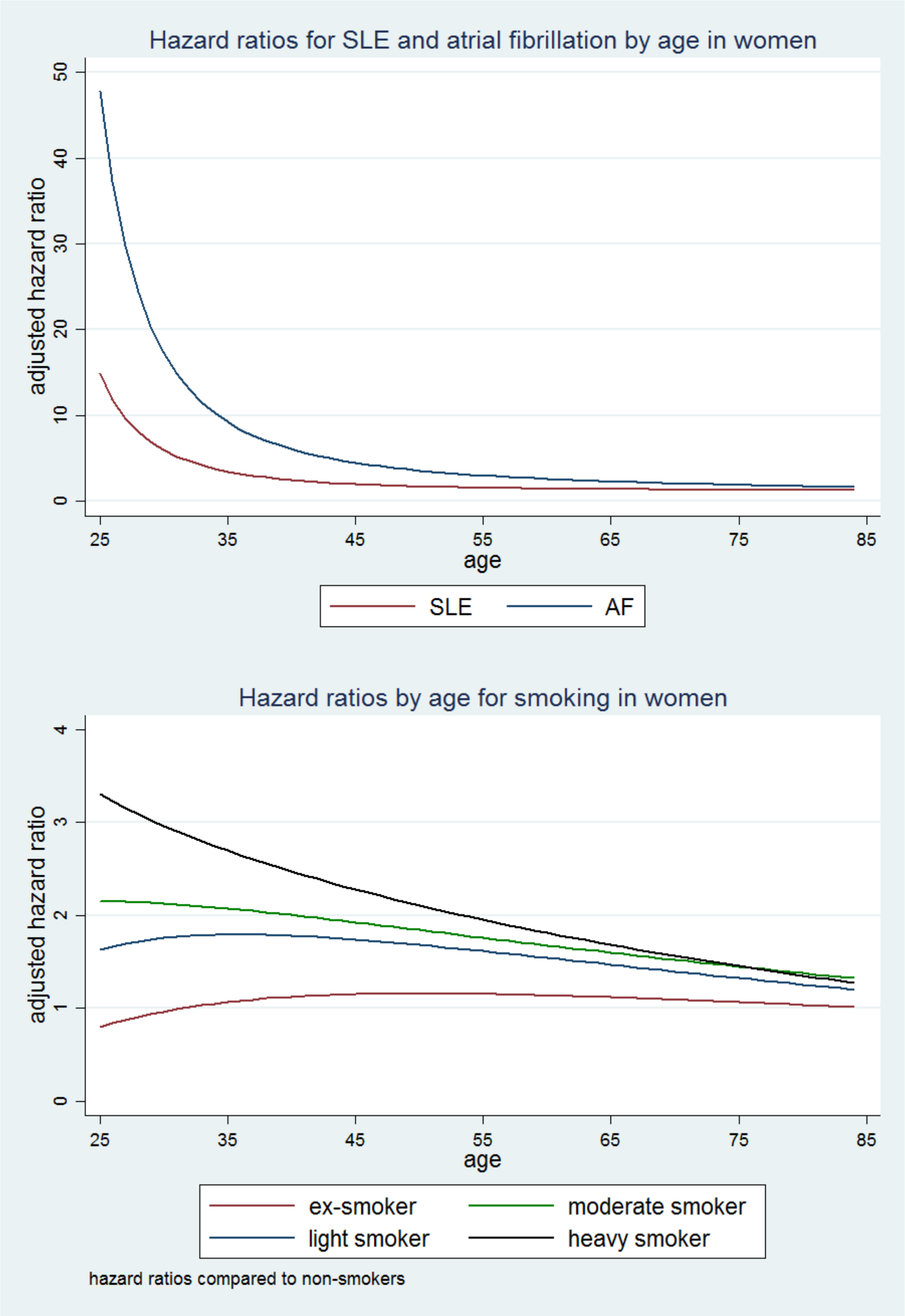
Hazard ratios for SLE and atrial fibrillation by age in women
adjusted hazard ratio
0
10
20
30
40
50
25
35
45
55
65
75
85
age
SLE
AF
Hazard ratios by age for smoking in women
adjusted hazard ratio
0
1
2
3
4
25
35
45
55
65
75
85
age
ex-smoker
moderate smoker
light smoker
heavy smoker
hazard ratios compared to non-smokers

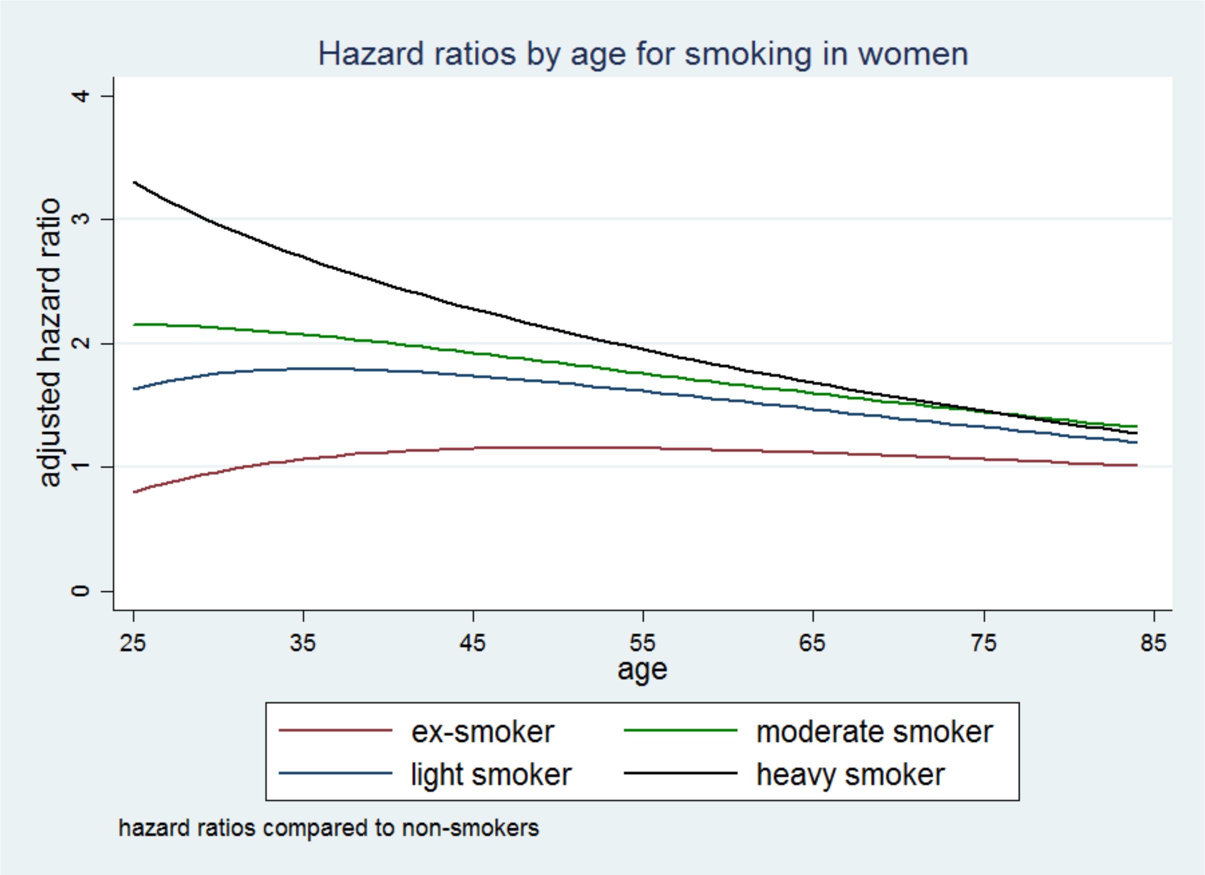
Hazard ratios by age for smoking in women
adjusted hazard ratio
0
1
2
3
4
25
35
45
55
65
75
85
age
ex-smoker
moderate smoker
light smoker
heavy smoker
hazard ratios compared to non-smokers

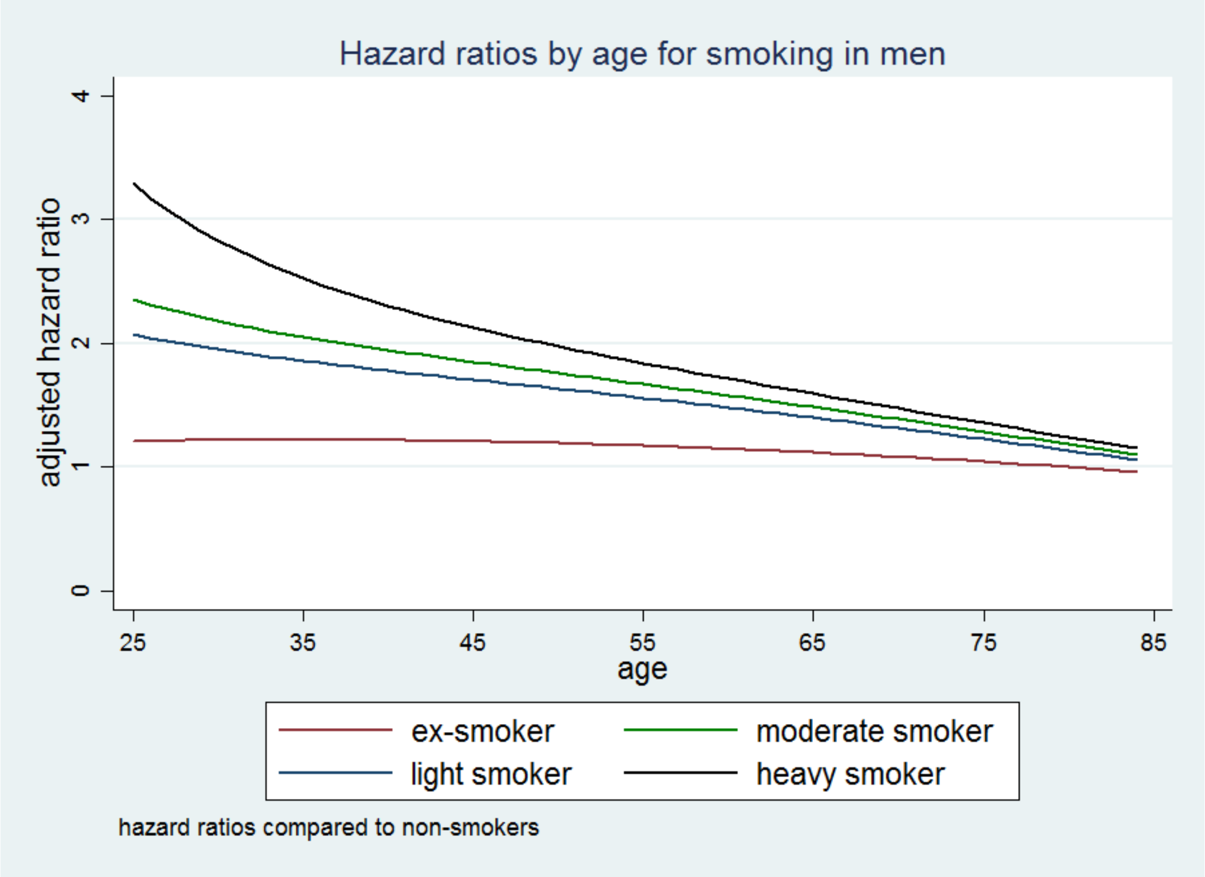
Hazard ratios by age for smoking in men
adjusted hazard ratio
0
1
2
3
4
25
35
45
55
65
75
85
age
ex-smoker
moderate smoker
light smoker
heavy smoker
hazard ratios compared to non-smokers

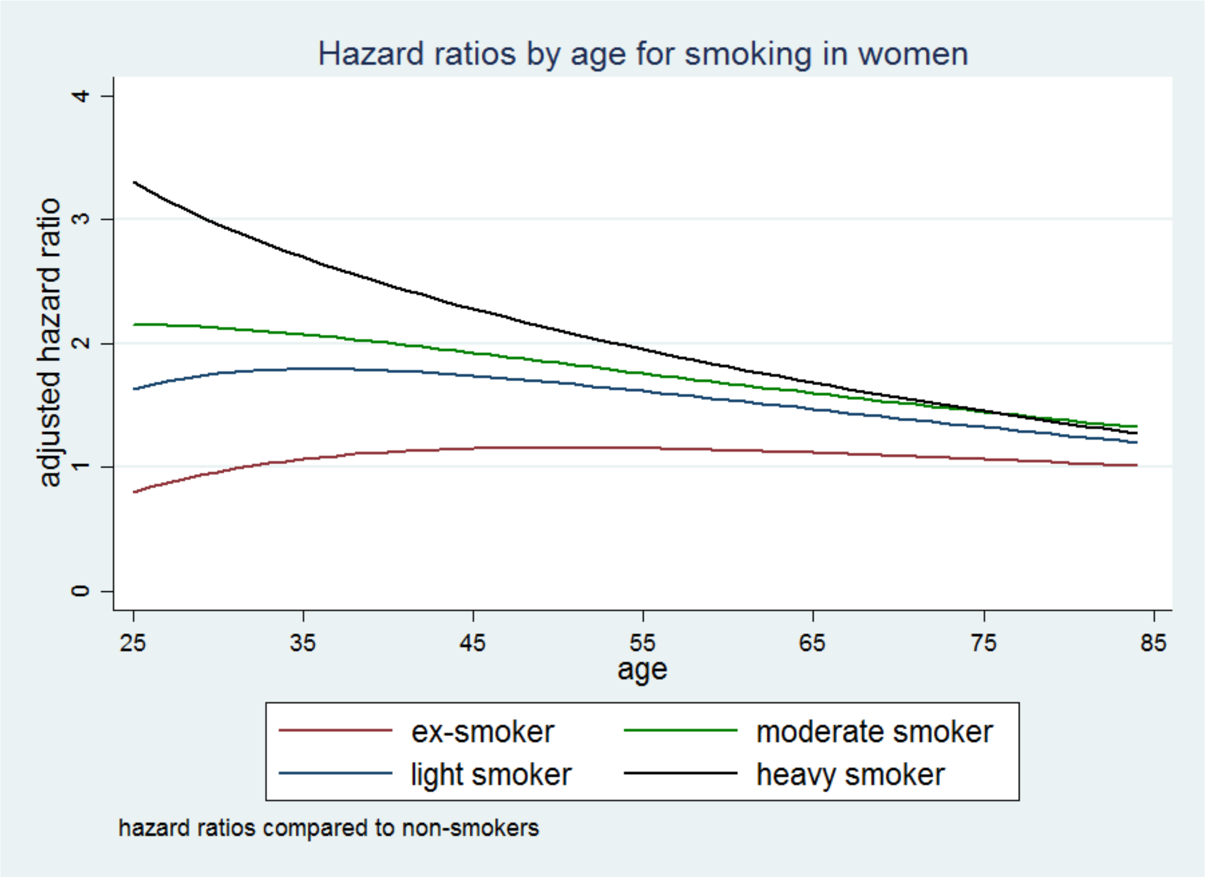
Hazard ratios by age for smoking in women
adjusted hazard ratio
0
1
2
3
4
25
35
45
55
65
75
85
age
ex-smoker
moderate smoker
light smoker
heavy smoker
hazard ratios compared to non-smokers

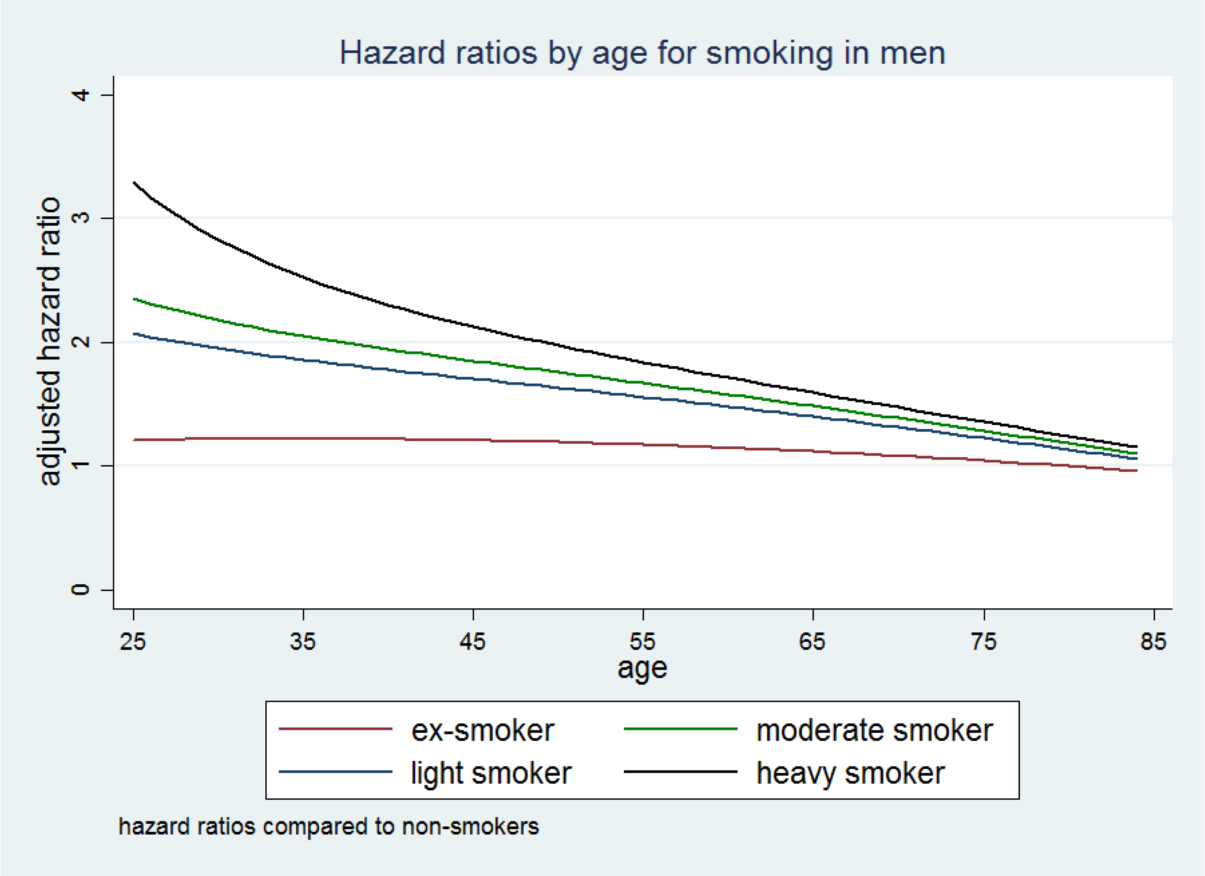
Hazard ratios by age for smoking in men
adjusted hazard ratio
0
1
2
3
4
25
35
45
55
65
75
85
age
ex-smoker
moderate smoker
light smoker
heavy smoker
hazard ratios compared to non-smokers
